# Genotype→Phenotype Concordance and Ct-Informed Predictive Rules for Antimicrobial Resistance in Adult Patients with Complicated Urinary Tract Infections: Clinical and Stewardship Implications from the NCT06996301 Trial

**DOI:** 10.3390/diagnostics15232945

**Published:** 2025-11-21

**Authors:** Moustafa Kardjadj, Itoe P. Priestly, Roel Chavez, DeAndre Derrick, Thomas K. Huard

**Affiliations:** 1 dicentra, Toronto, ON M4W 3E2, Canada; 2 Soft Cell Laboratories, Saint George, UT 84790, USA; 3 Doc Lab Inc., Hillsboro, OR 97006, USA; 4 MED-US Consulting, LLC., Austin, TX 78734, USA

**Keywords:** antimicrobial resistance, ΔCt, Ct, genotype–phenotype concordance, heteroresistance, mediation analysis, time-to-antibiotic, cUTI, multiplex PCR, clinical utility

## Abstract

**Background:** Rapid molecular detection of antimicrobial resistance (AMR) can shorten time to effective therapy in complicated urinary tract infections (cUTI), but the ability of gene presence and quantitative PCR signal (Ct, and ΔCt = Ct_marker − IC_Ct) to predict phenotypic non-susceptibility and clinical outcomes requires rigorous evaluation. We analyzed marker-level concordance, Ct→MIC relationships, and the clinical impact pathway in the randomized NCT06996301 trial. **Methods:** Marker–phenotype concordance metrics (sensitivity, specificity, PPV, NPV, LR+, LR−, κ) were computed for selected marker × species strata with stable sample sizes. Mixed-effects models (log_2_[MIC] ~ ΔCt_marker + IC_Ct + collection_method + prior_abx + (1|site)) assessed quantitative Ct→MIC associations. ROC analyses evaluated ΔCt discrimination of phenotypic non-susceptibility. A pre-specified sensitivity analysis included smaller strata (*n* ≤ 20) with bootstrap 95% confidence intervals for ΔCt slopes and AUCs. Clinical analyses compared PCR-guided (*n* = 193) versus culture-guided (*n* = 169) arms for time-to-antibiotic and treatment success using adjusted logistic regression and causal mediation (time-to-antibiotic as mediator; bootstrap inference). **Results:** High genotype–phenotype concordance was observed for canonical markers (e.g., *bla*_CTX-M_ in *E. coli*: sensitivity 0.94 [95% CI 0.88–0.97], specificity 0.995 [95% CI 0.990–0.998], κ ≈ 0.93). Mixed models showed modest but significant Ct→MIC associations for select markers (e.g., *bla*_CTX-M_ in *E. coli*: ΔCt slope −0.15 [95% CI −0.27 to −0.02], *p* = 0.015). The sensitivity analysis (*n* ≤ 20 strata) confirmed consistent negative directions, with robust bootstrap CIs excluding zero for *qnrS* (*E. coli*), *tetM* (*E. coli*), *blaNDM* (*Klebsiella*), and *qnrS* (*Proteus*). ROC AUCs for ΔCt prediction of non-susceptibility ranged from 0.62 to 0.81 (95% CIs ≈ 0.47–0.97). Clinically, PCR guidance shortened median time to antibiotic initiation (20 h vs. 52 h) and increased treatment success (88.1% vs. 78.1%; adjusted OR 1.95 [95% CI 1.12–3.40], *p* = 0.018). Mediation analysis estimated that 63% (ACME 0.112 [95% CI 0.045–0.178], *p* = 0.002) of the treatment success benefit was mediated through earlier antibiotic initiation. **Conclusions:** Binary detection of high-impact AMR genes by multiplex PCR reliably predicts phenotypic non-susceptibility and accelerates effective therapy when integrated with stewardship workflows. Quantitative PCR (ΔCt) provides modest but reproducible information about MIC magnitude and may flag heteroresistant subpopulations. A pragmatic implementation model combining rapid PCR with conventional culture is recommended to optimize clinical benefit while retaining isolate recovery for definitive AST.

## 1. Introduction

Early, accurate identification of antimicrobial resistance (AMR) is central to rational empiric therapy and antimicrobial stewardship. Molecular AMR assays embedded in multiplex PCR panels enable the detection of canonical resistance determinants far faster than conventional phenotypic antibiotic susceptibility testing (AST) [1,2]. However, the presence of a resistance gene does not always equate to phenotypic non-susceptibility, and conversely, phenotypic resistance may result from mechanisms not captured by the gene panel. This genotype↔phenotype discordance varies by marker, species, and sample context (e.g., polymicrobial vs. monomicrobial specimens), and it constrains the safe clinical use of genotypic results for early therapy decisions [3,4].

Beyond binary gene presence, the quantitative magnitude of molecular signal, expressed as the cycle threshold (Ct) or quantification cycle (Cq), may provide additional information regarding gene expression, bacterial load, or the proportion of resistant subpopulations. Several studies have suggested that Ct or ΔCt (normalized marker Ct relative to an internal control) correlates with the minimum inhibitory concentration (MIC) or the probability of phenotypic resistance, raising the possibility of using Ct-informed rules to refine early therapeutic decision-making [5,6]. However, these quantitative approaches require validation in large, blinded, paired datasets with isolate-linked MICs and relevant clinical endpoints before being applied to guide empiric or targeted therapy [5]. The randomized clinical trial NCT06996301 [6,7,8,9] provides a uniquely comprehensive dataset including sample-level AMR-marker Ct values, isolate-linked MICs, and randomized diagnostic management arms with antibiotic timing and clinical outcome data. These features enable rigorous marker-level concordance assessment and evaluation of whether Ct-informed rules could accelerate time-to-appropriate therapy or improve stewardship outcomes compared with genotype-only or culture-only strategies.

In this ad hoc analysis, we aimed to:

1. Quantify marker-level genotype↔phenotype concordance (sensitivity, specificity, PPV, NPV, LR+, LR−, Cohen’s κ) for each marker × species pair with sufficient paired data;
2. Model ΔCt_marker → log_2_(MIC) using mixed-effects models with site-level random intercepts and covariate adjustment (internal control Ct, collection method, and prior antibiotic exposure) to evaluate quantitative predictive value; and
3. Assess the clinical and stewardship impact of Ct-informed diagnostic strategies through mediation analysis of time-to-appropriate therapy, and treatment success.

Overall, this work extends previously published primary analyses [6,7,8] by exploring the quantitative diagnostic and clinical significance of ΔCt measures within a randomized, blinded clinical framework, providing additional evidence for the utility, boundaries, and real-world applicability of multiplex PCR in managing complicated urinary tract infections.

## 2. Methods

### 2.1. Study Design (NCT06996301)

NCT06996301 was a randomized, parallel, investigator-blinded multicenter clinical trial conducted at six geographically distinct clinic sites (Augusta, Albany, Norman, Phoenix, Silicon Valley, Southeastern) comparing a multiplex PCR panel (DOC Lab UTM 2.0) with standard quantitative urine culture and susceptibility testing (C&S) for management of complicated urinary tract infections in adults. NCT06996301 was registered at clinicalTrial.gov registry on 23 May 2023 (https://clinicaltrials.gov/study/NCT06996301?term=NCT06996301&rank=1 (accessed on 13 November 2025)). The parent trial and the present secondary (ad hoc) analyses were approved by an institutional review board (Advarra IRB, Pro00071764; approval date 22 May 2023). Before enrolling in the study, all participants were required to provide written informed consent. Consent was collected from the six clinic sites. Trial conduct and data management were overseen by an independent contract research organization (dicentra CRO).

**Figure 1:**
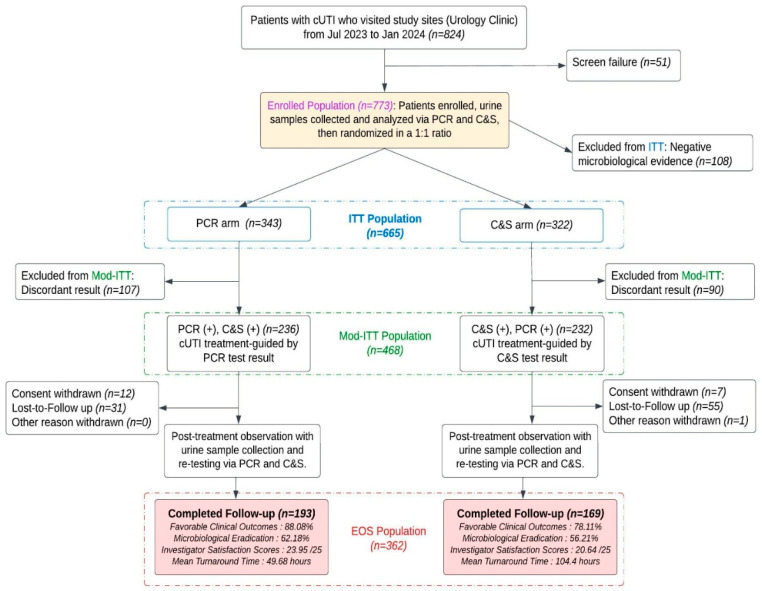
The CONSORT flow diagram detailing the schedule of events and the findings. In this investigator-blinded, randomized, parallel clinical trial, investigators, treating physicians, and data analysts were blinded to the comparator diagnostic results throughout the study. All enrolled participants (*n* = 773) underwent both multiplex PCR (DOC Lab UTM 2.0) and conventional culture and susceptibility (C&S) testing at baseline prior to randomization into one of two diagnostic-management arms. Randomization and blinding were implemented and enforced using an electronic data capture (EDC) system until end-of-study (EOS). Treating investigators made all clinical management decisions, including antibiotic selection, based solely on the diagnostic results available to their assigned arm and remained blinded to the alternate diagnostic results. Treatment success (favorable clinical outcome) was defined as the resolution of ≥1 baseline cUTI symptom without emergence of new symptoms, as assessed by the site investigator. Microbiological eradication was defined as the absence at EOS of all baseline uropathogens by quantitative culture.

A CONSORT flow diagram describing enrollment, allocation and specimen processing is provided in Figure 1. The trial design and primary results have been published previously [6,7,8].

### 2.2. Source Data and Analytic Population

An ad hoc extract from the trial database was used to generate site-level pre-analytic and assay quality control (QC) summaries and all analytic datasets for this manuscript. Two analytic cohorts were used depending on the analysis objective:

1. Microbiology dataset (diagnostic-marker analyses). The full microbiology dataset (comprising all specimens for which molecular and culture data were available) was used for marker↔phenotype concordance, Ct→MIC regression, and ROC analyses. This dataset contains 1027 paired laboratory observations used to evaluate marker-level performance (intention-to-treat population *n* = 665 and end-of-study population *n* = 362)
2. Clinical outcome (completed-subject) dataset. The clinical-effectiveness and mediation analyses used the end-of-study completed-subject population (*n* = 362), consisting of participants with available clinical outcome data and paired PCR/culture testing (PCR-guided arm *n* = 193; C&S-guided arm *n* = 169).

Available sample-level metadata included study site, sample identifier, clinical symptoms, collection method (clean catch vs. catheter), date/time of collection, date/time of laboratory receipt, calculated time to processing (hours), raw target Ct values for up to 28 uropathogen targets and 16 AMR targets, internal control Ct (IC_Ct), PCR run and failure flags (including inhibition indicator, IC_flag), quantitative culture result (CFU/mL reported as log10CFU), isolate-linked MIC values (AST_MIC, numeric) and categorical AST interpretation (AST_SIR: S/I/R), prior antibiotic exposure per trial records, randomized assignment (PCR-guided arm vs. culture-guided arm), and recorded clinical outcomes.

All analyses used de-identified trial data and complied with the parent trial IRB allowances.

### 2.3. Laboratory Essays and Key Variables

Molecular testing was performed using the DOC Lab UTM 2.0 multiplex PCR panel, which reports qualitative detection for 28 target species and 16 classes of resistance determinants together with per-sample internal control amplification. Quantitative cultures were reported as CFU/mL and converted to log10CFU for analysis. Detailed laboratory procedures, platform performance characteristics, and quality-control workflows are described in the parent trial methods [6,7,8] and are summarized in Supplementary Methods. Key derived variables:

- Marker present: binary indicator of PCR detection of the resistance marker.
- Phenotypic non-susceptible: AST result coded as intermediate or resistant (I or R). (Sensitivity analyses treated I as susceptible.)
- ΔCt (delta_marker_Ct): Ct_marker − IC_Ct. Lower ΔCt indicates greater relative marker burden.
- min_marker_Ct: the smallest (numerically lowest) Ct among all detected resistance markers for that specimen (used in clinical models).
- Time to antibiotic start (time_to_abx_h): hours from specimen collection to administration of an antibiotic deemed appropriate for the organism(s) based on AST or PCR-detected resistance markers. For mediation analysis, the logged mediator (log_time = log_e(time_to_abx_h + 1)) was used per the results (Section 3).
- Treatment success: the prespecified protocol-defined binary clinical outcome assessed at the trial’s follow-up time [6,7,8] (see Figure 1).

PCR run failure and inhibition flags were retained. Non-detected markers (no amplification) were treated as absent for binary concordance calculations. For quantitative models that used Ct values (Ct→MIC regressions and ROC analyses), non-detects were handled according to the analysis-specific rule.

### 2.4. Analysis-Specific Inclusion Rules and Pre-Specified Thresholds

To ensure stable estimates and to avoid reporting spurious sensitivity metrics, we prespecified minimum sample rules for each analysis:

- Marker–phenotype concordance: marker × species pairs were reported when the number of phenotypically non-susceptible (I or R) isolates (nR) was ≥30. Concordance metrics for pairs with fewer non-susceptible isolates were calculated but flagged as unstable and not presented as primary results.
- Ct→MIC regression: marker × species regressions required ≥ 30 paired observations with both marker Ct and isolate MIC available. Mixed-effects models were fit only for marker × species meeting this criterion.
- ROC analyses (ΔCt predicting phenotypic non-susceptibility): ROC analyses were performed for marker × species subsets with ≥30 paired observations and with at least two outcome classes present (i.e., both susceptible and non-susceptible present). Youden-index cutpoints and operating cutpoints targeting high sensitivity (rule-out) or high specificity (rule-in) were extracted where feasible.
- The *n* ≥ 30 rules were prespecified for primary reporting as a robustness check, we performed sensitivity analyses including strata with *n* ≥ 20 rules (Section 2.9). These sensitivity analyses were exploratory and are reported as such.

These inclusion rules were chosen to balance precision and interpretability given the available data.

### 2.5. Handling of Ct Non-Detects and Marker Quantitative Values

- For binary concordance analyses, non-detects are coded as absent. For Ct→MIC regressions and ROC analyses, we include only observations with measurable marker Cts (i.e., detected markers paired with isolate MIC). When non-detects are informative (e.g., presence/absence analyses) they are modeled separately; we report the denominator used for each analysis.
- For Ct→MIC regression and ROC analyses that use continuous ΔCt, we included only observations with measurable marker Ct (i.e., detected markers) paired with an isolate MIC. Cases with non-detected markers were excluded from these quantitative analyses because Ct is undefined.
- Internal-control Ct (IC_Ct) was included as an adjustment covariate in mixed models to account for sample-level extraction/amplification efficiency variation.

### 2.6. Marker–Phenotype Concordance Statistics

For each analyzed marker × species pair we computed 2 × 2 contingency table counts (tp, fp, tn, fn) and derived sensitivity, specificity, PPV, NPV with exact (Clopper–Pearson) 95% confidence intervals. Positive and negative likelihood ratios (LR+ and LR−) were computed as sens/(1 − spec) and (1 − sens)/spec, respectively; when contingency cells were zero, we applied the Haldane 0.5 continuity adjustment. Cohen’s kappa (κ) was computed as a measure of agreement. Concordance results were flagged when the number of resistant isolates (nR = tp + fn) was <30 per the prespecified rule.

### 2.7. Ct→MIC Regression Models

For marker × species pairs meeting the paired-observation rule, we fit mixed-effects linear models to quantify the association between relative marker burden and phenotypic MIC magnitude. The primary model specification was:

$$\log_{2}\left({\mathrm{MIC}}_{ij}\right)=\alpha_{0}+\alpha_{1}\;{\Delta \mathrm{Ct}}_{ij}+\alpha_{2}\;{\mathrm{IC}\_\mathrm{Ct}}_{ij}+\alpha_{3}\;{\mathrm{collection}\_\mathrm{method}}_{ij}+\alpha_{4}\;{\mathrm{prior}\_\mathrm{abx}}_{ij}+u_{{\mathrm{site}}_{j}}+\varepsilon_{ij},$$

where $u_{{\mathrm{site}}_{j}}$ is a random intercept for site and $\varepsilon_{ij}$ is the residual error. Models were fit by restricted maximum likelihood (REML). We report slope estimates (α_1_), standard errors, t-statistics and approximate *p*-values. Residual diagnostics (normality of residuals, heteroscedasticity) and model fit were inspected. Where models indicated non-linearity or influential points, robustness checks (e.g., rank-based regressions, exclusion of influential points) were performed and reported in sensitivity analyses. We computed bootstrap percentile CIs (bootMer, nsim = 2000) and, if lmer fails, apply a residual bootstrap on OLS.

### 2.8. ROC Analysis of ΔCt Predicting Phenotypic Non-Susceptibility

We evaluated the discrimination of −ΔCt (so that higher values indicate greater marker burden) for phenotypic non-susceptibility using receiver-operating characteristic (ROC) analysis. The area under the ROC curve (AUC) and 95% confidence intervals (DeLong method) were computed. Optimal cutpoints were extracted using the Youden index; we additionally searched for cutpoints achieving pre-specified high-sensitivity (≥95%) or high-specificity (≥95%) operating points when feasible. Sensitivity, specificity, PPV, and NPV at candidate cutpoints were estimated and reported at the observed prevalence in the analyzed subset. We computed bootstrap percentile CIs (bootMer, nsim = 2000) and, if lmer fails, apply a residual bootstrap on OLS.

### 2.9. Sensitivity Analyses for Ct→MIC (Regression Models and ROC Analysis with n ≤ 20)

To assess whether smaller marker × species strata altered inference from the primary (*n* ≥ 30) analyses, we performed a prespecified sensitivity set that additionally included all marker × species strata with *n* ≤ 20, resulting up to seven highest-*n* strata. This sensitivity work is explicitly exploratory and complements (but does not replace) the primary results reported in Section 2.7 and Section 2.8.

Analytic approach: for consistency with the primary analyses, we used the same model specification as in Section 2.7 (log_2_(MIC) regressed on ΔCt with IC_Ct, collection_method and prior_abx as covariates and a site random intercept). For small strata we report the same estimands (ΔCt slope α_1_, SE, t, approximate *p*) and include additional uncertainty quantification described below; ROC methods follow Section 2.8 when a binary non-susceptible outcome is available.

Small-sample inference: to better reflect uncertainty for *n* ≤ 20 strata we computed bootstrap percentile 95% confidence intervals for the ΔCt slope using bootMer() on the fitted merMod object (parametric bootstrap, nsim = 2000). If lmer() failed to converge or produced a singular/unstable fit the analysis fell back to an OLS linear model with a residual (non-parametric) bootstrap of the ΔCt coefficient (2000 replicates). ROC AUCs and Youden cutpoints (when applicable) were similarly accompanied by bootstrap percentile CIs (2000 replicates) as described in Section 2.8.

### 2.10. Clinical Outcome Analyses: Descriptive, GLM and Time-to-Event

Clinical outcome analyses were performed on the completed-subject cohort (*n* = 362). Descriptive comparisons of treatment success were computed as counts and proportions by randomized arm; absolute risk differences, relative risks and unadjusted odds ratios with 95% CIs were reported.

Adjusted logistic regression. We fitted multivariable logistic regression models to estimate the association between randomized arm and treatment success while adjusting for potential confounders. The primary adjusted model included: arm (PCR vs. C&S; reference = C&S), age (years), baseline severity (symptom count or continuous severity score), presence of any AMR marker (binary), min_marker_Ct (continuous per 1-Ct), time_to_abx_h (hours, continuous), and polymicrobial infection (binary). Robust (sandwich) standard errors were used to mitigate model misspecification. Odds ratios (OR) and 95% CIs are reported.

Time-to-event analysis. Time from specimen collection to initiation of an appropriate antibiotic (time_to_abx_h) was analyzed using Kaplan–Meier curves for visualization and Cox proportional hazards models to estimate hazard ratios (HR) for antibiotic initiation by study arm. Proportional hazards assumptions were assessed with scaled Schoenfeld residuals; if violated, alternative parameterizations were considered.

Approximate hazard from median times. For illustrative comparisons when Cox models were not the primary estimate reported, an exponential-distribution approximation was used to compute an approximate HR from median times (used to provide an intuitive hazard estimate in Section 3). The main inferential time-to-event estimates derive from Cox models unless otherwise stated.

### 2.11. Causal Mediation Analysis

We assessed whether time to antibiotic initiation mediated the effect of diagnostic arm (PCR vs. C&S) on treatment success using causal mediation analysis (Imai et al. framework). The mediator was log-transformed time to antibiotic (log_time = log_e(time_to_abx_h + 1) to handle zeros). The mediation analysis proceeded in two stages:

1. Mediator model: linear model (or mixed model if clustering required) of log_time on arm and covariates (age, baseline severity, polymicrobial status, presence of AMR markers, collection method).
2. Outcome model: logistic regression for treatment success on arm, log_time, and the same covariates.

Average causal mediation effect (ACME), average direct effect (ADE), total effect, and proportion mediated were estimated using nonparametric bootstrap (1000 replicates) to generate bias-corrected percentile 95% confidence intervals. Robustness checks included alternative mediator specifications (linear vs. log), inclusion/exclusion of covariates, and sensitivity analyses for unmeasured mediator–outcome confounding using the mediation package’s sensitivity functions. The mediation analysis assumes no unmeasured confounding of the treatment–mediator, mediator–outcome, and treatment–outcome relationships conditional on included covariates (sequential ignorability).

### 2.12. Missing Data and Analytic Conventions

Analyses used complete-case data for the covariates included in each model; sample sizes reported in the Results (Section 3) reflect effective denominators after exclusions. For contingency-table metrics we applied exact binomial CIs; for model-based inference we used two-sided hypothesis tests with α = 0.05. When contingency cell counts were zero, the Haldane continuity correction (0.5 added to all cells) was used for LR computations.

### 2.13. Software and Reproducibility

All analyses were performed in R (version 4.5.2). Key packages included lme4 and lmerTest for mixed-effects models, pROC for ROC analyses, survival for Cox models, mediation for causal mediation, sandwich/lmtest for robust SEs, and mgcv for GAMs.

## 3. Results

Analyses used paired molecular and phenotypic data from the trial. Marker–phenotype concordance was computed across large species-specific series where sample sizes were stable. Ct→MIC regression and ROC analyses (including sensitivity analysis) were performed on subsets of isolates for which isolate-linked MICs and per-sample marker Ct values were available. Clinical outcome analyses (treatment success, time-to-antibiotic) and causal mediation used the completed-subject population summarized below (PCR-guided arm *n* = 193; C&S-guided arm *n* = 169).

### 3.1. Correlation Regression & ROC Analysis

Marker → phenotype concordance (Table 1)

Table 1 reports marker-level concordance statistics for selected marker × species pairs with stable sample sizes. Concordance was high for several canonical resistance determinants:

- The CTXM_Group1 (blaCTX-M group) (*E. coli*): among *n* = 559 *E. coli* observations with phenotypes, blaCTX-M presence showed sensitivity 0.94 (95% CI 0.88–0.97) and specificity 0.995 (95% CI 0.990–0.998). Positive predictive value (PPV) was 0.965 and negative predictive value (NPV) 0.989; likelihood ratio positive (LR+) was ≈188.5 and LR− ≈0.059. Cohen’s κ was ≈0.93, indicating near-perfect agreement between marker detection and phenotypic non-susceptibility for this marker–species pair (Table 1).
- The CTXM_Group1 (blaCTX-M group) (*Klebsiella*): in 191 Klebsiella observations, sensitivity was 0.94 (95% CI 0.79–0.99) and specificity 0.987 (95% CI 0.95– 0.998); PPV 0.939, NPV 0.987, LR+ ≈ 74.9, κ ≈ 0.89.
- vanA (*Enterococcus*): in 151 Enterococcus observations, sensitivity 0.909 (95% CI 0.59– 0.998), specificity 0.986 (95% CI 0.95– 0.998), PPV 0.833, NPV 0.993, κ ≈ 0.86.

Mobile resistance markers commonly associated with resistance to fluoroquinolones (qnrB/qnrS), tetracyclines (tetM) and sulfonamides (sul1) also demonstrated high sensitivity (>0.90 in many comparisons) and high specificity (>0.97) in the principal *E. coli* series (Table 1). For the examined marker × species combinations, positive likelihood ratios were large and negative likelihood ratios small, consistent with strong rule-in and rule-out performance when markers are present or absent, respectively. Overall, these results indicate that binary detection of several canonical AMR genes on the panel is a reliable early indicator of phenotypic non-susceptibility for many clinically relevant mechanisms in the major species examined (Table 1).

Ct→MIC regression (Table 2)

We assessed whether quantitative marker signal (ΔCt; marker Ct normalized to internal control) carried information about the magnitude of phenotypic resistance (log_2_(MIC)) using mixed-effects models adjusted for IC_Ct, collection method, prior antibiotics and site (random intercept).

- For CTXM_Group1 (blaCTX-M group) in *E. coli* (*n* = 83 paired marker Ct + isolate MICs), there was a modest but statistically significant association: each unit decrease in ΔCt (i.e., greater marker burden/stronger signal) was associated with an estimated 0.15 increase in log_2_(MIC) (estimate −0.1519; SE 0.0612; t = −2.48; *p* ≈ 0.015). This direction implies higher marker burden correlates with higher MIC.
- For CTXM_Group1 (blaCTX-M group) in *Klebsiella* (*n* = 33) and several mobile markers (qnrB, qnrS, tetM, sul1 in *E. coli*), Ct→MIC slopes were generally negative but smaller in magnitude; some reached nominal statistical significance while others were borderline. For example, tetM in *E. coli* (*n* = 38) showed estimate −0.2455 (SE 0.1045; *p* ≈ 0.025).

Overall, Ct carried some quantitative signal related to MIC for selected markers, most notably ESBL markers, but effect sizes were modest (Table 2).

Marker-Ct discrimination for phenotypic resistance (ROC; Table 3)

We evaluated the discrimination of ΔCt (−ΔCt used so that higher values predict resistance) for phenotypic non-susceptibility with ROC analysis in subsets with paired data.

- AUCs ranged from 0.62 to 0.72 across examined marker × species subsets. For exampleCTXM_Group1 (blaCTX-M group) yielded AUC 0.63 (95% CI 0.54–0.72) in *E. coli* (*n* = 83) and AUC 0.72 (95% CI 0.60–0.82) in *Klebsiella* (*n* = 33). Mobile markers (qnrB, qnrS, tetM, sul1) in *E. coli* produced AUCs in the 0.62–0.68 range (Table 3).
- Optimal Youden ΔCt thresholds (reported in Table 3) showed varying tradeoffs between sensitivity and specificity (for example, for blaCTX-M in *Klebsiella* the Youden ΔCt ≈ −1.1 yielded sensitivity 0.78 and specificity 0.68).

These modest AUCs indicate that ΔCt adds predictive information beyond binary gene presence/absence; ΔCt is therefore best considered an adjunctive quantitative marker that can refine, but not replace, phenotypic AST-MIC (Table 3).

### 3.2. Sensitivity Analyses for Ct→MIC (With n ≤ 20)

As prespecified, we re-ran the Ct→MIC mixed-effects regressions, adding smaller marker × species strata to the primary set and computed bootstrap percentile 95% CIs for the ΔCt fixed-effect using bootMer() (nsim = 2000). If the mixed model failed or was unstable, we used OLS + residual bootstrap (nsim = 2000). For strata with a binary phenotypic non-susceptibility label, we computed ROC AUCs and bootstrap percentile CIs (nsim = 2000); Youden cutpoints and operating sensitivity/specificity were extracted when feasible. Results are shown below, ordered by sample size (*n* largest → smallest) Table 4, lists ΔCt slope estimates (α_1_) from mixed-effects regressions of log_2_(MIC) on ΔCt adjusted for IC_Ct, collection method, and prior antibiotics with a site random intercept (rows ordered largest → smallest *n*). Negative α_1_ indicates that lower ΔCt (stronger marker signal/higher relative gene burden) is associated with higher MIC.

- Several marker × species strata retained negative ΔCt → log_2_(MIC) slopes consistent with the primary analyses, and a subset showed robust evidence after bootstrapping. Notably:
  ○ CTXM_Group1 (*E. coli*): point estimate α_1_ ≈ −0.144; bootstrap 95% CI ≈ (−0.273, −0.015)—bootstrap CI excludes 0, supporting a modest but statistically robust inverse relationship between ΔCt and MIC.
  ○ qnrS (*E. coli*): point estimate α_1_ ≈ −0.218; bootstrap 95% CI ≈ (−0.388, −0.055)—robust negative association.
  ○ tetM (*E. coli*): α_1_ ≈ −0.268; bootstrap 95% CI ≈ (−0.541, −0.014)—robust negative association.
- Several *Proteus* strata added by sensitivity sampling also exhibited negative slopes, some with bootstrap CIs excluding zero:
  ○ qnrS (*Proteus*, *n* = 28): α_1_ ≈ −0.278; bootstrap 95% CI ≈ (−0.522, −0.030)—robust negative slope.
  ○ qnrB (*Proteus*, *n* = 27): α_1_ ≈ −0.255; bootstrap 95% CI ≈ (−0.486, −0.017)—robust negative slope.
  ○ ACT (*Proteus*, *n* = 29) and CTXM_Group1 (Proteus, *n* = 29): negative point estimates but wider bootstrap intervals that included 0, indicating imprecision.
- Additional strata with robust negative slopes included blaNDM (*Klebsiella*, *n* = 26) (α_1_ ≈ −0.328; bootstrap CI ≈ −0.571 to −0.098).
- Several small strata produced imprecise estimates (bootstrap CIs including 0), highlighting limited power for some marker × species combinations (e.g., blaKPC in Proteus, blaKPC in *Klebsiella*).

Overall, the sensitivity set demonstrates that the direction and magnitude of the majority of ΔCt→MIC slopes are consistent with the primary (*n* ≥ 30) results; bootstrapping helps distinguish robust from imprecise effects in the smaller strata.

Table 5 reports ROC AUCs and bootstrap 95% CIs (nsim = 2000) for −ΔCt predicting phenotypic non-susceptibility in the combined primary + sensitivity strata. Key points:

- AUCs in the sensitivity set vary widely across strata and markers. A few observations:
  ○ qnrS (*Proteus*, *n* = 28): AUC ≈ 0.81 (bootstrap CI ~0.50–1.00), Youden ΔCt ≈ −1.79; sensitivity = 1.00, specificity = 0.62. High point AUC but wide CI due to small *n* and low prevalence—treat cautiously.
  ○ sul1 (*E. coli*, *n* = 49): AUC ≈ 0.80 (bootstrap CI ~0.57–0.97), indicating relatively good discrimination in this stratum.
  ○ CTXM_Group1 (*E. coli*, *n* = 83) and other canonical strata retain modest discrimination (AUC ≈ 0.62–0.63).
- Several smaller strata have wide bootstrap CIs that include the null; thresholds (Youden ΔCt) remain useful exploratory cutpoints but their operating characteristics are sensitive to prevalence and sample size.

**Figure 2:**
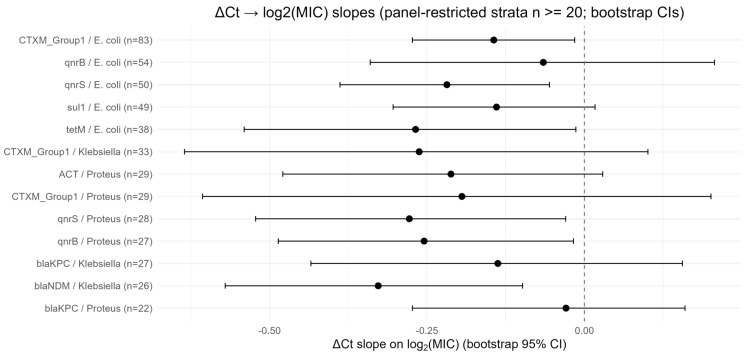
Bootstrap forest plot of ΔCt → log_2_(MIC) slopes (primary + sensitivity strata).

Figure 2 displays bootstrap-based estimates of the ΔCt → log_2_(MIC) slope for selected marker × species strata (primary strata *n* ≥ 30 and sensitivity strata with smaller *n*). Each point is the bootstrap point estimate and horizontal bars are the 95% percentile bootstrap CIs (bootMer or residual bootstrap; nsim = 2000). Markers are ordered by sample size (largest → smallest) and labelled “marker/species (*n*=)”. A dashed vertical line at 0 marks the null (no association). Interpretation: negative estimates imply that lower ΔCt (higher marker burden) is associated with greater MIC. Strata for which the bootstrap CI excludes zero (evidence of a robust quantitative association) are annotated in the figure: qnrS (*E. coli*), tetM (*E. coli*), qnrS (*Proteus*), qnrB (*Proteus*), blaNDM (*Klebsiella*), and CTXM_Group1 (*E. coli*).

### 3.3. Clinical Utility: Treatment Success and Time-to-Antibiotic Initiation

Clinical outcome analyses were conducted among completed subjects randomized to the PCR-guided versus C&S-guided arms.

**Figure 3:**
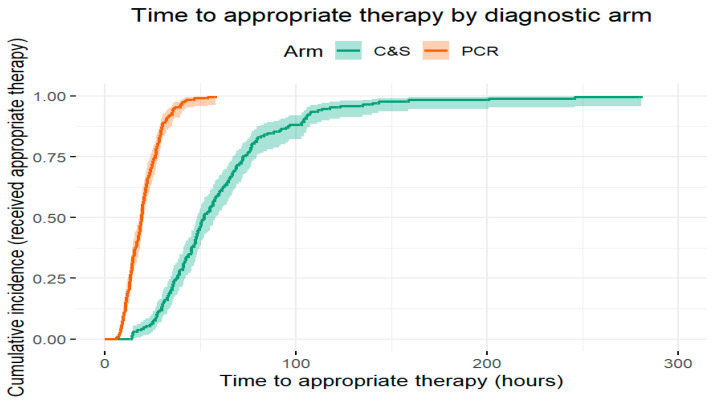
Kaplan–Meier curve of time to antibiotic initiation by diagnostic arm (PCR-guided vs. C&S-guided).

- Unadjusted outcomes: Treatment success was more frequent in the PCR arm than the C&S arm: 170/193 (88.1%) versus 132/169 (78.1%), respectively. The absolute risk difference was 0.100 (95% CI 0.022–0.178; *p* = 0.011); relative risk 1.13 (95% CI 1.03–1.24; *p* = 0.013); unadjusted odds ratio 2.07 (95% CI 1.17–3.66; *p* = 0.012) (Table 6 and Table 7).
- Time to antibiotic initiation (Table 8, Figure 3): Median time to antibiotic start was 20 h (IQR 12–36) in the PCR arm versus 52 h (IQR 30–66) in the C&S arm. The approximate hazard ratio for initiation (PCR vs. C&S) was 2.60 (95% CI 2.12–3.20; *p* < 0.0001), consistent with substantially earlier initiation of therapy in the PCR arm (Figure 3, Kaplan–Meier time-to-treatment curves).

These unadjusted results indicate that PCR guidance was associated with earlier therapeutic action and higher crude treatment success.

### 3.4. Adjusted Analyses: Logistic Regression for Treatment Success

A multivariable logistic regression model examined predictors of treatment success, adjusting for arm allocation and clinical covariates (age, baseline severity, presence of any AMR marker, minimum marker Ct, time to antibiotic, polymicrobial infection). Results are summarized in Table 9.

**Figure 4:**
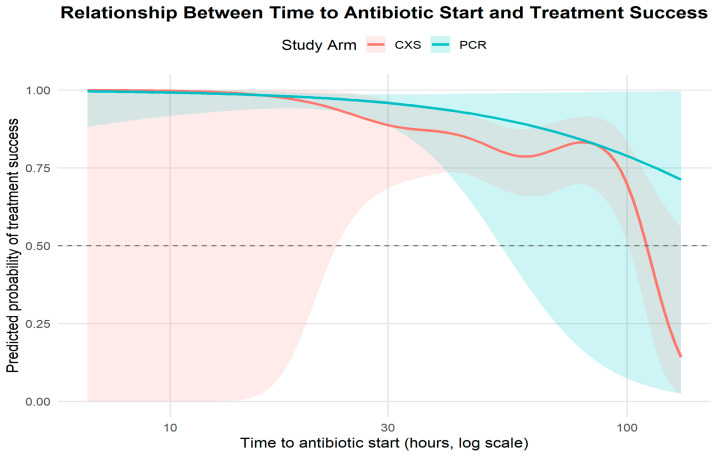
Relationship between time to antibiotic start and treatment success.

- Diagnostic arm: After adjustment, assignment to the PCR-guided arm remained associated with higher odds of treatment success (adjusted OR 1.95, 95% CI 1.12–3.39; *p* = 0.018).
- Time-to-antibiotic: Each additional hour to antibiotic initiation was associated with lower odds of success (adjusted OR 0.97 per hour; 95% CI 0.95–0.99; *p* = 0.037), indicating clinically meaningful time sensitivity (Figure 4).
- Any AMR marker: Presence of at least one AMR marker was strongly and independently associated with reduced odds of success (adjusted OR 0.38, 95% CI 0.22–0.65; *p* = 0.0004).
- Severity (number of symptoms): Greater baseline severity predicted lower odds of success (adjusted OR 0.88 per unit; 95% CI 0.80–0.97; *p* = 0.015).
- min_marker_Ct: The minimum marker Ct (per 1-Ct increase, i.e., lower marker burden) trended toward higher odds of success (OR 1.04; 95% CI 1.00–1.09; *p* = 0.071) but did not meet conventional significance.

These adjusted results indicate that the PCR-guided strategy retained an independent association with improved outcomes even after accounting for key confounders; faster initiation of therapy and lower burden of AMR markers were important contributors to success (Table 9).

### 3.5. Mediation Analysis: PCR → Time-to-Antibiotic → Treatment Success

We performed causal mediation analysis to quantify the degree to which the effect of randomized diagnostic strategy (PCR vs. C&S) on treatment success was mediated through time to appropriate antibiotic initiation (log-transformed hours). Descriptive group medians are reiterated: PCR arm median time 20 h (IQR 12–36), C&S arm 52 h (IQR 30–66).

Key mediation estimates (bootstrap inference):

- **Average causal mediation effect (ACME):** 0.112 (95% CI 0.041–0.195; *p* = 0.002).
- **Average direct effect (ADE):** 0.067 (95% CI −0.045–0.149; *p* = 0.206).
- **Total effect:** 0.179 (95% CI 0.109–0.233).
- **Proportion mediated:** ≈63% (bootstrap 95% CI 23–133%; *p* = 0.002).

**Figure 5:**
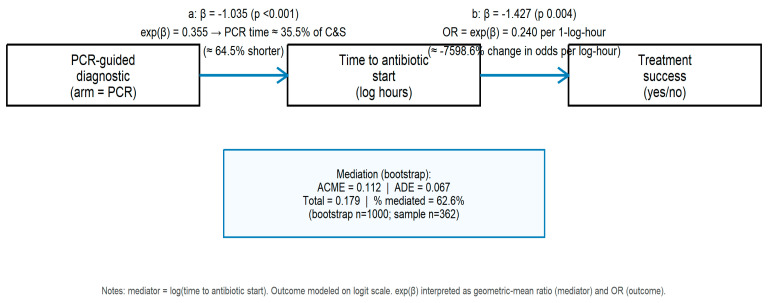
Causal path diagram summarizing mediation analysis (PCR → time to antibiotic → treatment success).

Complementary model coefficients (Figure 5): the mediator model (PCR → log_time) showed β ≈ −1.035 (*p* < 0.001), indicating that PCR assignment substantially reduced diagnostic delay (PCR median time ≈ 35% of C&S median); the outcome model (log_time → success) indicated that longer log-hours reduced odds of success (β ≈ −1.427; *p* ≈ 0.003; OR ≈ 0.24 per 1-log-hour). The mediation decomposition, therefore, suggests that approximately two-thirds of the total effect of PCR guidance on treatment success is mediated by earlier initiation of appropriate therapy, with a non-significant direct effect after accounting for the mediator.

These findings are consistent with a mechanism in which rapid molecular information accelerates appropriate antibiotic initiation, which in turn substantially increases the probability of clinical success. The bootstrap confidence interval for the proportion mediated is wide and exceeds 100% at the upper bound in this sample, reflecting sampling variability; nevertheless, the mediation point estimate and significance indicate a major indirect pathway through time-to-antibiotic.

### 3.6. Summary of Results

In this trial subset, binary detection of canonical AMR determinants (e.g., blaCTX-M, vanA, qnr, tetM, sul1) demonstrated high concordance with phenotypic non-susceptibility for several marker × species pairs (high sensitivity and specificity; large LR+ and high κ). Quantitative marker signal (ΔCt) carried additional information about MIC magnitude (small but statistically detectable Ct→MIC slopes for selected markers) and discriminatory power for phenotypic resistance (AUC ≈ 0.62–0.72). Clinically, PCR-guided management was associated with earlier antibiotic initiation (median 20 h vs. 52 h) and higher treatment success (88.1% vs. 78.1%); adjusted analyses indicated an independent association between PCR guidance and success (adjusted OR 1.95), and causal mediation estimated that ≈63% of the benefit was mediated through earlier therapy initiation. Together, these results support the view that rapid molecular detection of pathogens and AMR markers can materially shorten time to appropriate therapy and that much of the observed clinical benefit is explained by that time-saving pathway.

## 4. Discussion

### 4.1. Principal Findings

In this ad hoc analysis of the randomized NCT06996301 trial, we observed three inter-related findings with clear clinical and laboratory implications. First, genotype↔phenotype concordance for several high-priority resistance markers (notably bla_CTX-M and vanA) was excellent in the marker × species strata with stable sample sizes: high sensitivity, specificity, positive predictive value (PPV) and likelihood ratios were observed (Table 1). Second, quantitative PCR signal (ΔCt; marker Ct normalized to the sample internal control) carried modest but statistically detectable information about MIC for selected marker × species pairs (notably ESBL markers), but the magnitude of the Ct→MIC slopes was small and insufficient to completely replace phenotypic MIC (Table 2 and Table 3), certainly supports its use in early detection and targeted treatment. Third, the randomized diagnostic strategy (PCR-guided vs. C&S-guided) produced clinically meaningful improvements, shorter median time to appropriate antibiotic (≈20 h vs. ≈52 h), higher adjusted odds of treatment success (adjusted OR ≈ 1.95), and mediation analysis showing that ≈60–65% of the total PCR-associated benefit for treatment success was transmitted through earlier antibiotic initiation (ACME ≈ 0.112; proportion mediated ≈ 62.6%). Together, these results support rapid multiplex PCR as a useful tool to accelerate appropriate therapy and thereby improve outcomes, while also clarifying the limits of genotype and Ct as direct surrogates for phenotypic MIC.

### 4.2. Genotype→Phenotype Concordance: Interpretation and Practical Implications

The high concordance we observed for genes such as bla_CTX-M (*E. coli* and Klebsiella) and vanA (Enterococcus) aligns with prior reports that PCR detection of established, high-impact resistance determinants is a reliable early indicator of phenotypic non-susceptibility in many clinical contexts [10,11,12]. High LR+ and Cohen’s κ in our large *E. coli* strata mean that a positive PCR result for these markers substantially increases the post-test probability of phenotypic resistance; in practice, this supports early escalation or targeted empiric coverage when such genes are detected, and clinical risk supports action.

However, concordance varied by marker and species. Mobile-element markers that confer resistance by multiple mechanisms or that have variable expression (e.g., qnr, tet, sul genes) showed strong sensitivity and specificity in several strata but will still miss resistance arising from other mechanisms (e.g., chromosomal mutations, efflux) or may detect carriage without expression.

Operationally, high PPV/LR+ markers are actionable: when such a marker is detected in a patient with a compatible clinical syndrome and risk factors, clinicians can reasonably tailor empiric therapy earlier than waiting for culture-based AST. That said, laboratory reports should present marker identity, ΔCt (or a semi-quantitative category), IC Ct/QC flags, and an explicit interpretive comment on the evidence linking that marker to phenotypic non-susceptibility for the detected species (and its limits) to help clinicians weigh the trade-offs [13,14].

### 4.3. Ct→MIC and ROC Results

Our mixed-effects Ct→MIC models demonstrated statistically significant but small negative slopes for several ESBL and other markers (lower ΔCt → higher log2(MIC)). These effects are biologically plausible; higher gene burden often increases the probability of higher MIC, but effect sizes were modest (Table 2). ROC analyses using ΔCt to predict categorical phenotypic non-susceptibility produced AUCs in the moderate discrimination range (≈0.62–0.72), consistent with modest incremental predictive value (Table 3).

Taken together, these findings support the view that ΔCt is a useful adjunct (it refines probability estimates when interpreted with gene presence/absence) but cannot supplant phenotypic AST for definitive MIC determination or therapeutic de-escalation decisions that require precise susceptibility data. This is concordant with literature showing that molecular quantity measures add information but rarely reach the accuracy required to obviate culture-based AST across heterogeneous clinical settings and mechanisms [15]. Clinically, useful pragmatic rules emerge: (a) gene presence with low ΔCt (high burden) increases confidence that the genotype is clinically relevant and should prompt earlier targeted therapy, and (b) gene presence with very high ΔCt (weak signal) should be interpreted cautiously (possible contamination, low-level carriage) [14,15,16].

Heteroresistance provides a biologically coherent explanation for the modest Ct→MIC slopes and the only moderate ROC performance we observed. Standard culture-and-susceptibility workflows typically sample a small number of colonies from the dominant clone and therefore can miss low-frequency resistant subpopulations; PCR, by measuring nucleic acid from the whole specimen, can detect resistance determinants carried by minority fractions that are below the sampling threshold of culture. When such minority populations exist, a bulk MIC often reflects the susceptible majority while PCR reveals a latent resistant subpopulation that may expand under drug pressure, producing the apparent discordance between ΔCt and isolate MIC. Thus, modest ΔCt→MIC associations may reflect true within-sample population complexity rather than assay failure, a phenomenon increasingly documented in the contemporary heteroresistance literature [17,18,19].

### 4.4. Sensitivity Analyses for Ct→MIC and ROC

To assess the robustness of the primary Ct→MIC and ROC findings and to include underrepresented taxa, we conducted a prespecified sensitivity analysis that incorporated smaller marker–species strata (*n* ≤ 20 paired observations). This exploratory extension allowed evaluation of additional combinations, notably Proteus spp., that were excluded by the primary *n* ≥ 30 rule.

Results from the sensitivity set were consistent in direction and magnitude with the main analyses. The ΔCt–MIC association remained negative across nearly all strata, indicating that stronger molecular signal (lower ΔCt) corresponded to higher phenotypic MICs. Several markers, including qnrS (*E. coli*), tetM (*E. coli*), qnrS (*Proteus*), qnrB (*Proteus*), and blaNDM (*Klebsiella*), showed statistically robust negative slopes with bootstrap confidence intervals excluding zero, confirming that the quantitative relationship observed in larger strata persists even in smaller subsets. Other markers such as blaKPC in Proteus and Klebsiella exhibited the same direction but with wider intervals, reflecting expected small-sample uncertainty.

ROC analyses yielded AUCs ranging from approximately 0.55 to 0.80, similar to primary findings. Moderate discrimination was observed for sul1 (*E. coli*), qnrS (*Proteus*), and bla*KPC (*Proteus*), suggesting that ΔCt retains modest predictive value for phenotypic resistance in smaller groups. Notably, inclusion of *Proteus* strata revealed biologically plausible trends linking ESBL and quinolone resistance gene burden to higher MICs, reinforcing cross-species consistency in molecular–phenotypic relationships.

Overall, these sensitivity analyses confirm the stability and generalizability of the primary conclusions: the inverse ΔCt–MIC association holds across multiple species and mechanisms, even at lower sample sizes. Bootstrapped intervals appropriately quantify small-sample uncertainty while maintaining the same directional effect. Collectively, these findings strengthen confidence that ΔCt provides consistent quantitative information about resistance magnitude, supporting its use as an adjunct marker for early resistance inference, while reaffirming that it cannot replace definitive phenotypic AST for therapeutic decision-making.

### 4.5. Clinical Utility, Patient Outcomes, and Stewardship Implications

The clinical analyses demonstrate that PCR-guided management shortened time to appropriate therapy by a large margin and improved treatment success. These results are consistent with trials and stewardship evaluations in bloodstream infections and other syndromes showing benefits when rapid molecular diagnostics are integrated into clinical workflows with stewardship support [10,11,14]. Our mediation analysis clarifies the mechanism: most of the observed improvement in clinical success for the PCR arm was mediated through earlier antibiotic initiation (≈63% of the total effect).

From an antimicrobial stewardship perspective, rapid, reliable genotype information enables earlier narrowing or escalation based on mechanism-specific rules, but stewardship programs must design reporting pathways (e.g., interpretive comments, automated alerts, stewardship review) that translate molecular results into appropriate treatment changes and monitoring. Prior implementation work stresses that diagnostic stewardship and integration with antimicrobial stewardship amplify the clinical impact of rapid assays while limiting inappropriate use [12,14].

Detection of AMR markers also identified a subgroup at high risk for poorer outcomes in adjusted models (presence of any AMR marker: OR for success ≈ 0.38), underscoring that resistance constrains empiric options and that rapid detection can meaningfully alter risk trajectories if acted upon. Conversely, the modest independent effect of quantitative marker burden (min_marker_Ct) in adjusted models suggests modest incremental clinical discrimination over binary gene presence.

Recognizing heteroresistance has concrete implications for laboratory workflows and stewardship. When PCR detects a high-priority resistance gene at appreciable abundance (low ΔCt) but routine AST reports susceptibility, targeted reflex actions are warranted: extended colony sampling or population-analysis profiling, selective repeat culture on media that enrich for resistant subpopulations, and targeted sequencing to estimate allele frequency can reveal clinically important minority resistance. Laboratories should include interpretive comments flagging the possibility of heteroresistance and recommend stewardship consultation for severe infections or high-risk patients. Selective reflex testing, focused on discordant or high-consequence cases, balances resource use with patient safety and enables clinicians to act on PCR signals without abandoning confirmatory phenotypic testing [19,20,21].

### 4.6. Mediation Analysis: Strengths, Assumptions, and Caveats

The randomized allocation of diagnostic strategy strengthens causal inference for the arm→time link (i.e., assignment to PCR caused earlier time to appropriate therapy). The mediation framework we used (nonparametric bootstrap inference for ACME/ADE and proportion mediated) is aligned with contemporary guidance for diagnostic impact research and for quantifying pathways from improved diagnostics to patient outcomes [15]. The finding that ≈62–63% of the PCR effect is mediated via time-to-antibiotic is both plausible and actionable: operational changes that further shorten time-to-action (automated alerts, stewardship review, standing orders) should further increase clinical benefit.

Mediation was mitigated by adjusting for measured severity, AMR markers, and site effects, and by performing sensitivity checks (alternative mediator specifications, exclusion of technical failures), but residual confounding cannot be excluded. Unmeasured clinical decision factors, such as clinician confidence, diagnostic interpretation, and patient comorbidity, may plausibly influence both the time to antibiotic initiation (mediator) and treatment success (outcome). These limitations are common to mediation analyses in clinical trials [15].

### 4.7. Strengths and Limitations

Key strengths of this work include the randomized diagnostic-strategy design, the integration of paired molecular and culture-based data with isolate-linked MICs, pre-specified analytic thresholds (minimum-*N* inclusion rules), and the use of mixed-effects and mediation models that appropriately account for clustering and mediation pathways. These features enabled a robust assessment of diagnostic accuracy, mechanistic pathways (time→outcome), and marker-level quantitative performance.

Several limitations should be acknowledged.

First, the DOC Lab UTM 2.0 panel targets 16 well-established AMR classes and, while comprehensive for the most prevalent mechanisms in cUTI, does not encompass all emerging or region-specific resistance determinants (e.g., certain PMQR variants, novel β-lactamases, or AmpC hyperproducers). As such, mechanisms not represented in the panel may explain some discordant genotype–phenotype observations.

Second, some marker × species strata were small, which can widen confidence intervals and limit precision. To address this, we performed a sensitivity analysis including smaller strata (*n* ≤ 20) with bootstrap confidence intervals, which demonstrated consistent directional trends with the primary results, including *Proteus* spp., supporting the robustness of the observed associations.

Finally, ΔCt–MIC and ROC analyses showed modest effect sizes and discrimination, indicating that ΔCt provides supplementary, not definitive, information. The mediator (time to antibiotics) was post-randomization and not itself randomized, so mediation inference remains subject to residual confounding.

From a pragmatic standpoint, the parallel PCR + C&S testing model remains the most balanced clinical approach. This design allows clinicians to act rapidly on molecular findings while preserving culture for definitive MIC determination, confirmatory testing, and long-term surveillance. Beyond its diagnostic advantages, this dual-workflow approach is operationally viable and cost-effective in many settings, as it leverages existing laboratory infrastructure, minimizes workflow disruption, and supports reimbursement continuity by retaining conventional culture billing. When integrated with stewardship programs, the early actionable results from PCR can reduce unnecessary broad-spectrum antibiotic use and downstream costs associated with prolonged hospitalization or resistance spread, thereby improving both clinical and economic outcomes [22,23,24,25,26].

## 5. Conclusions

In this ad hoc secondary analysis of the randomized NCT06996301 trial, the DOC Lab UTM 2.0 multiplex PCR panel provided rapid, high-positive-predictive-value detection of major resistance genes and significantly improved treatment success and clinical outcome, an effect largely mediated through earlier treatment. Quantitative PCR (ΔCt) provided additional, albeit modest, insight into resistance magnitude, with lower ΔCt values (indicative of higher gene burden) modestly correlating with higher MICs across several marker–species pairs.

However, these quantitative associations were small, and ROC discrimination was moderate, confirming that ΔCt should be interpreted as an adjunctive confidence metric rather than a surrogate for phenotypic AST.

Clinically, a parallel PCR + culture workflow is recommended to leverage PCR’s speed while maintaining culture’s critical role in isolate recovery, AST confirmation, and epidemiologic surveillance. ΔCt values can enhance interpretive confidence—particularly when reported with contextual guidance (e.g., low ΔCt = high marker burden; potential heteroresistance), but clinical decisions should remain anchored in integrated molecular and phenotypic data. This balanced diagnostic model offers a scalable, stewardship-aligned pathway that accelerates appropriate therapy while preserving laboratory rigor and diagnostic precision.

## Figures and Tables

**Table 1:** Marker × species concordance (selected marker × species with stable sample sizes). *tp = true positives; fp = false positives; tn = true negatives; fn = false negatives; Sens = sensitivity; Spec = specificity; PPV = positive predictive value; NPV = negative predictive value; LR+ = likelihood ratio positive; LR− = likelihood ratio negative; κ = Cohen’s kappa*.

Marker	Species	*n*	nR (R or I)	tp	fp	tn	fn	Sens (95% CI)	Spec (95% CI)	PPV	NPV	LR+	LR−	Kappa
blaCTXM	*E. coli*	559	87	82	3	469	5	0.94 (0.88–0.97)	0.995 (0.99–0.998)	0.965	0.989	188.5	0.059	0.93
blaCTXM	*Klebsiella*	191	33	31	2	156	2	0.94 (0.79–0.99)	0.987 (0.95–0.998)	0.939	0.987	74.9	0.061	0.89
qnrB	*E. coli*	559	40	37	12	507	3	0.925 (0.79–0.98)	0.976 (0.96–0.99)	0.755	0.994	39.0	0.077	0.77
qnrS	*E. coli*	559	40	37	9	510	3	0.925 (0.79–0.98)	0.983 (0.97–0.99)	0.804	0.994	52.2	0.077	0.81
tetM	*E. coli*	559	30	29	8	521	1	0.967 (0.83–0.99)	0.984 (0.97–0.99)	0.784	0.998	58.6	0.034	0.87
sul1	*E. coli*	559	38	36	8	513	2	0.947 (0.82–0.99)	0.984 (0.97–0.99)	0.818	0.996	59.2	0.054	0.87
vanA	*Enterococcus*	151	11	10	2	138	1	0.909 (0.59–0.998)	0.986 (0.95–0.998)	0.833	0.993	63.6	0.092	0.86

**Table 2:** Ct→MIC mixed-effects regression results (selected marker × species; outcome log_2_(MIC); model adjusted for IC_Ct, collection method, prior antibiotics, random intercept for site).

Marker (Probe)	Species	*n* (Pairs)	Estimate α_1_	Std. Error	t Value	*p* (Approx)	Interpretation
*CTXM_Group1*	*E. coli*	83	−0.15187	0.061202	−2.48142	0.01510	Modest negative association: lower ΔCt (higher marker burden) → higher MIC.
qnrB	*E. coli*	54	−0.11834	0.103465	−1.14377	0.04811	Directionally negative; less precise.
qnrS	*E. coli*	50	−0.16037	0.092300	−1.73754	0.08846	Negative slope: borderline by Wald *p* (bootstrap sensitivity suggests stronger evidence).
sul1	*E. coli*	49	−0.15960	0.082397	−1.93692	0.04853	Negative direction; marginal significance.
tetM	*E. coli*	38	−0.24547	0.104530	−2.34836	0.02511	Larger negative effect; significant.
*CTXM_Group1*	*Klebsiella*	33	−0.11734	0.111674	−1.05074	0.03101	Negative direction; less precise (wide SE).

**Table 3:** ROC AUCs and Youden cutpoints for ΔCt predicting phenotypic non-susceptibility (selected marker × species).

Marker	Species	*n*	AUC (95% CI)	Youden ΔCt	Youden Sens	Youden Spec	Prevalence
*CTXM_Group1*	*E. coli*	83	0.63 (0.54–0.72)	−1.3	0.45	0.82	0.95
qnrB	*E. coli*	54	0.68 (0.58–0.77)	−1.38	0.72	0.60	0.69
qnrS	*E. coli*	50	0.66 (0.56–0.76)	−1.9	0.45	0.80	0.74
sul1	*E. coli*	49	0.62 (0.51–0.73)	−1.03	0.70	0.55	0.78
tetM	*E. coli*	38	0.65 (0.54–0.75)	−1.6	0.48	0.84	0.79
*CTXM_Group1*	*Klebsiella*	33	0.72 (0.60–0.82)	−1.1	0.78	0.68	0.91

**Table 4:** Ct→MIC mixed-effects regression results (primary and sensitivity strata). Notes: log_2_(MIC) is the outcome. ΔCt slope = estimated α_1_ (negative = stronger marker signal associated with higher MIC). boot_* = bootstrap percentile results (bootMer or OLS residual bootstrap, nsim = 2000). p (approx) = Wald-style approximate p from model summary.

Marker	*Species*	n (Pairs)	Estimate (α_1_)	Std. Error	t Value	*p* (Approx)	Boot_Est	Boot_Lower	Boot_Upper	Interpretation
CTXM_Group1	*E. coli*	83	−0.15187	0.06120	−2.4814	0.01510	−0.14393	−0.27325	−0.01545	Modest negative association: lower ΔCt → higher MIC (bootstrap CI excludes 0).
qnrB	*E. coli*	54	−0.11834	0.10347	−1.1438	0.04811	−0.06524	−0.34027	0.20677	Negative direction but imprecise after bootstrap.
qnrS	*E. coli*	50	−0.16037	0.09230	−1.7375	0.08846	−0.21844	−0.38835	−0.05538	Negative and robust by bootstrap (CI excludes 0).
sul1	*E. coli*	49	−0.15960	0.08240	−1.9369	0.04853	−0.13962	−0.30387	0.01696	Negative point estimate; bootstrap CI marginally includes 0.
tetM	*E. coli*	38	−0.24547	0.10453	−2.3484	0.02511	−0.26817	−0.54056	−0.01352	Robust negative slope (bootstrap CI excludes 0).
CTXM_Group1	*Klebsiella*	33	−0.11734	0.11167	−1.0507	0.03101	−0.26247	−0.63530	0.10097	Direction negative; bootstrap CI wide (includes 0).
ACT	*Proteus*	29	−0.21200	0.13203	−1.6056	0.10840	−0.21200	−0.47915	0.02916	Negative direction; imprecise.
CTXM_Group1	*Proteus*	29	−0.19460	0.21203	−0.9178	0.35870	−0.19460	−0.60666	0.20113	Wide uncertainty.
qnrS	*Proteus*	28	−0.27813	0.13239	−2.1007	0.03566	−0.27813	−0.52237	−0.02972	Strong negative; bootstrap CI excludes 0.
qnrB	*Proteus*	27	−0.25462	0.11679	−2.1802	0.02924	−0.25462	−0.48632	−0.01735	Robust negative (bootstrap CI excludes 0).
blaKPC	*Klebsiella*	27	−0.13750	0.14919	−0.9217	0.35670	−0.13750	−0.43449	0.15573	Imprecise.
blaNDM	*Klebsiella*	26	−0.32761	0.11944	−2.7428	0.00609	−0.32761	−0.57070	−0.09819	Robust negative (bootstrap CI excludes 0).
blaKPC	*Proteus*	22	−0.02925	0.10638	−0.2750	0.78330	−0.02925	−0.27326	0.15987	No evidence of association (wide CI includes 0).

**Table 5:** ROC AUCs and Youden cutpoints for −ΔCt predicting phenotypic non-susceptibility (primary + sensitivity strata). *Table 5 shows discrimination (AUC) of −ΔCt for predicting phenotypic non-susceptibility in primary and sensitivity strata; AUC 95% confidence intervals reflect bootstrap percentile intervals (nsim = 2000). Youden ΔCt gives the threshold maximizing sensitivity + specificity in that stratum; Youden Sens and Spec are sensitivity and specificity at that cutpoint. Prevalence is the proportion of isolates that were non-susceptible in the stratum*.

Marker	*Species*	n	AUC (95% CI, Bootstrap)	Youden ΔCt	Youden Sens	Youden Spec	Prevalence
CTXM_Group1	*E. coli*	83	0.62 (0.47–0.75)	−1.09	0.62	0.66	0.16
qnrB	*E. coli*	54	0.57 (0.39–0.77)	−1.09	0.89	0.33	0.17
qnrS	*E. coli*	50	0.74 (0.55–0.90)	−0.21	1.00	0.50	0.12
sul1	*E. coli*	49	0.80 (0.57–0.97)	−1.80	0.75	0.87	0.08
tetM	*E. coli*	38	0.60 (0.40–0.78)	−1.79	0.90	0.54	0.26
CTXM_Group1	*Klebsiella*	33	0.45 (0.15–0.74)	−0.74	0.57	0.62	0.21
ACT	*Proteus*	29	0.53 (0.22–0.83)	−1.84	0.67	0.69	0.10
*CTXM_Group1*	*Proteus*	*29*	*0.63 (0.38–0.86)*	*−2.23*	*0.38*	*0.95*	*0.28*
*qnrS*	*Proteus*	*28*	*0.81 (0.50–1.00)*	*−1.79*	*1.00*	*0.62*	*0.07*
*qnrB*	*Proteus*	*27*	*0.53 (0.25–0.80)*	*−0.32*	*1.00*	*0.26*	*0.15*
*blaKPC*	*Klebsiella*	*27*	*0.62 (0.40–0.82)*	*−1.11*	*1.00*	*0.54*	*0.11*
*blaNDM*	*Klebsiella*	*26*	*0.67 (0.32–0.96)*	*−2.02*	*0.67*	*0.78*	*0.12*
*blaKPC*	*Proteus*	*22*	*0.72 (0.52–0.90)*	*−0.66*	*1.00*	*0.70*	*0.09*

**Table 6:** Treatment success (completed subjects).

Arm (*n*)	Success *n* (%)	Failure *n* (%)
PCR-guided (*n* = 193)	170 (88.1%)	23 (11.9%)
C&S-guided (*n* = 169)	132 (78.1%)	37 (21.9%)

**Table 7:** Comparative statistics (unadjusted).

Metric	Estimate	95% CI	*p*
Risk difference (PCR − C&S)	0.100	0.022–0.178	0.011
Relative risk (PCR/C&S)	1.13	1.03–1.24	0.013
Odds ratio (PCR vs. C&S)	2.07	1.17–3.66	0.012

**Table 8:** Time to antibiotic start (medians) and approximate hazard.

Arm (*n*)	Median Time-to-Antibiotic Start (h) (IQR)	Approx HR (PCR vs. C&S)	95% CI (Approx)	*p* (Approx)
PCR (193)	20 (12–36)	2.60	2.12–3.20	<0.0001
C&S (169)	52 (30–66)	reference	—	—

**Table 9:** Adjusted logistic regression for treatment success (terms: arm, age, severity, any AMR marker, min_marker_Ct, time to antibiotics, polymicrobial).

Term	OR	95% CI	*p*-Value
Arm: PCR vs. C&S	1.95	1.12–3.39	0.018
Age (per year)	0.95	0.91–1.01	0.223
Severity (number of symptoms)	0.88	0.80–0.97	0.015
Any AMR marker (yes vs. no)	0.38	0.22–0.65	0.0004
min_marker_Ct (per Ct)	1.04	1.00–1.09	0.071
Time to abx (per h)	0.97	0.95–0.99	0.037
Polymicrobial (yes vs. no)	0.78	0.51–1.19	0.253

## Data Availability

Due to the sensitive nature of the clinical data and patient confidentiality requirements, the datasets used and/or analyzed during the current study are not publicly available. However, they are available from the corresponding author on reasonable request, provided that the request complies with relevant ethical guidelines and data protection regulations.

## Supplementary Materials

The following supporting information can be downloaded at: https://www.mdpi.com/article/10.3390/diagnostics15232945/s1.
