# Supplementary material for: Genotype→Phenotype Concordance and Ct-Informed Predictive Rules for Antimicrobial Resistance in Adult Patients with Complicated Urinary Tract Infections: Clinical and Stewardship Implications from the NCT06996301 Trial
Source: Diagnostics (Basel). 2025 Nov 21;15(23):2945. doi: 10.3390/diagnostics15232945 (PMC12691348; doi:10.3390/diagnostics15232945)
Supplement: Supplementary file 1 [file diagnostics-15-02945-s001.zip › Culture Report Sample.pdf]

Crisp Regional Hospital, Inc.
Harry S. Latham MD, Lab Director CLIA # 11D0646027
902 North 7th Street * Cordele, GA 31015

**Name:**	[redacted]		Admit Date:	
MRN / FIN:	[redacted]	[redacted]	Discharge Date:	
DOB / Age:	[redacted]	64 years	Location:	CRRH RAD
Sex / Birth Sex:	Female	Female	Ordering:	White,William K MD

Urinalysis

Legend: @=Abnormal !=Critical L=Low H=High c=Corrected f=Comment O=Order Comment i=Interp Data *=Performing Lab

UA Macroscopic

Procedure	Collected Date 6/20/2023<br>Collected Time 10:44 EDT	Units	Reference Range
Urine Srce	Clean Catch		
UA Color	Yellow		
UA Appear	Clear		
UA pH	6.0		
UA Spec Grav	1.020		[1.005-1.030]
UA Glucose	3+		
UA Bili	NEGATIVE		
UA Ketones	NEGATIVE		
UA Blood	1+		
UA Protein	3+		
UA Urobilinogen	0.2		
UA Nitrite	Negative		
UA Leuk Est	NEGATIVE		[>=0]
UA Micro Ind?	Indicated		
UA Culture Indicated?	Indicated		

UA Microscopic

Procedure	Collected Date 6/20/2023<br>Collected Time 10:44 EDT	Units	Reference Range
UA WBC	Moderate		
UA RBC	Rare		
UA Bacteria	Moderate		
UA Squam Epithelial	Rare		
UA Yeast	Present		
UA Fine Gran	Present		

**Name:**	[redacted]		Admit Date:	
MRN / FIN:	[redacted]	[redacted]	Discharge Date:	
DOB / Age:	[redacted]	64 years	Location:	CRRH RAD
Sex / Birth Sex:	Female	Female	Ordering:	White,William K MD

Microbiology

Legend: c=Corrected f=Comment *=Performing Lab S=Susceptible I=Intermediate R=Resistant N/A=Not Applicable

Procedure:	Urine Culture		
Source:	Urine	Accession:	13-23-171-0193
Collected Date/Time:	6/20/2023 10:44 EDT	Body Site:	
Start Date/Time:	6/20/2023 14:07 EDT	Free Text Source:	

FINAL REPORTS
Final Report
Verified Date/Time: 6/22/2023 08:59 EDT
>100,000 cfu/mL Escherichia coli

PRELIMINARY REPORTS
Preliminary Report
Verified Date/Time: 6/21/2023 09:40 EDT
>100,000 cfu/mL Gram Negative Bacilli
Identification and sensitivity to follow

SUSCEPTIBILITY RESULTS

	Escherichia coli	
Antibiotic	MIC Dilutn	MIC U Int
Amikacin	<=16	Susceptible
Ampicillin	>16	Resistant
Ampicillin/Sulbactam	>16/8	Resistant
Aztreonam	<=4	Susceptible
Cefazolin	<=2	Susceptible
Cefepime	<=2	Susceptible
Cefoxitin	<=8	Susceptible
Ceftazidime	<=1	Susceptible
Ceftriaxone	<=1	Susceptible
Ciprofloxacin	<=1	Susceptible
Ertapenem	<=0.5	Susceptible
Gentamicin	<=4	Susceptible
Imipenem	<=1	Susceptible
Levofloxacin	<=2	Susceptible
Meropenem	<=1	Susceptible
Nitrofurantoin	<=32	Susceptible
Tigecycline	<=2	Susceptible
Tobramycin	<=4	Susceptible
Trimethoprim/Sulfamethoxazole	<=2/38	Susceptible
