# Supplementary material for: Genotype→Phenotype Concordance and Ct-Informed Predictive Rules for Antimicrobial Resistance in Adult Patients with Complicated Urinary Tract Infections: Clinical and Stewardship Implications from the NCT06996301 Trial
Source: Diagnostics (Basel). 2025 Nov 21;15(23):2945. doi: 10.3390/diagnostics15232945 (PMC12691348; doi:10.3390/diagnostics15232945)
Supplement: Supplementary file 1 [file diagnostics-15-02945-s001.zip › PCR Report Sample.pdf]

UTI PCR Report

ALASKA UROLOGY

Final Report

Patient Information

Patient Name: UTI 5, UTI 5
Date of Birth: 8/7/1986
Age: 38 Years
Sex: Male

Provider Information

Ordering Physician: test
Lab Director: Ying Liu, M.D.
CLIA#: 02D0640642

Specimen Information

Accession ID: 250590120
Specimen Type: Urine
Collection Date: 02/28/2025
Result Date: 2/28/2025 1:05:25 PM

Result Summary

Organism(s) Tested - Detected:

Organism Detected	Est. Microbial Load*	Total % Pathogen Load	Potential Therapeutic Agents
Klebsiella pneumoniae/oxytoca	32.768*10^5 copies/mL	100.0000000%	- Linezolid<br>- Nitrofurantoin<br>- Cefiderocol<br>- Aztreonam<br>- Moxifloxacin<br>- Ofloxacin<br>- Piperacillin/Tazobactam<br>- Amoxicillin/Clavulanate (Augmentin) (PO)<br>- Amoxicillin/Clavulanate (Augmentin)<br>- Piperacillin/Tazobactam (IV)<br>- Ticarcillin/Clavulanate<br>- Cefazolin (Ancef)<br>- Cefazolin (Ancef) - 1st Gen<br>- Cefepime<br>- Gentamicin<br>- Amikacin<br>- Plazomicin<br>- Ceftriaxone - 3rd Gen<br>- Ceftriaxone<br>- Cefepime (IV) - 4th Gen<br>- Levofloxacin po/IV<br>- Levofloxacin<br>- Ciprofloxacin po/IV<br>- Ciprofloxacin<br>- Fosfomycin<br>- Colistin

Antibiotic Resistance Detected:

Resistance Gene Detected	Resistant Against
Class A ß-lactamase; blaKPC	Carbapenems
dfr (A1, A5), sul (1,2) probes (Sulfamethoxazole and trimethoprim)	Sulfonamides

UTI PCR Report

UTI 5, UTI 5   8/7/1986   Accession ID: 250590120

Medications:

	Klebsiella pneumoniae/oxytoca
Amikacin	S
Amikacin IV	-
Amoxicillin	-
Amoxicillin PO	-
Amoxicillin/Clavulanate (Augmentin)	S
Amoxicillin/Clavulanate (Augmentin) (PO)	S
Amphotericin B	-
Ampicillin	-
Ampicillin PO	-
Ampicillin/Sulbactam	-
Azithromycin	-
Azithromycin PO	-
Aztreonam	S
Cefazolin (Ancef)	S
Cefazolin (Ancef) - 1st Gen	S
Cefazolin (Ancef)	-
Cefdinir	-
Cefdinir PO - 3rd Gen	-
Cefepime	S
Cefepime (IV) - 4th Gen	S
Cefiderocol	S
Cefixime	-
Cefixime PO - 3rd Gen	-
Cefpodoxime	-
Cefpodoxime - 3rd Gen	-
Cefprozil	-
Ceftaroline fosamil (Teflaro)	-
Ceftazidime	-
Ceftazidime - 3rd Gen	-
Ceftriaxone	S
Ceftriaxone - 3rd Gen	S
Cefuroxime	-
Cephalexin (Keflex)	-
Cephalexin (Keflex) - 1st Gen	-
Cephalexin (Keflex)	-
Chloramphenicol	-
Ciprofloxacin	S
Ciprofloxacin po/IV	S
Clarithromycin	-
Clindamycin	-
Clindamycin PO	-

UTI PCR Report

UTI 5, UTI 5 8/7/1986 Accession ID: 250590120

	Klebsiella pneumoniae/oxytoca
Clotrimazole	-
Colistin	S
Dicloxacillin	-
Dicloxacillin PO	-
Doripenem	R
Doxycycline	-
Doxycycline po/IV	-
Ertapenem	R
Ertapenem IV	R
Erythromycin	-
Erythromycin PO	-
Fluconazole	-
Fluconazole po/IV	-
Flucytosine	-
Fosfomycin	S
Gemifloxacin	-
Gentamicin	S
Imipenem/Cilastatin	R
Imipenem/Cilastatin IV	R
Isavuconazole	-
Itraconazole	-
Ketoconazole	-
Levofloxacin	S
Levofloxacin po/IV	S
Linezolid	S
Linezolid PO	-
Meropenem	R
Meropenem IV	R
Metronidazole	-
Metronidazole (IV/po)	-
Miconazole	-
Minocycline	-
Moxifloxacin	S
Nafcillin	-
Nistatin	-
Nitrofurantoin	S
Nitrofurantoin (PO)	-
Ofloxacin	S
Oxacillin	-
Penicillin G	-
Piperacillin/Tazobactam	S
Piperacillin/Tazobactam (IV)	S
Plazomicin	S

UTI PCR Report

UTI 5, UTI 5 8/7/1986 Accession ID: 250590120

	Klebsiella pneumoniae/oxytoca
Posaconazole	-
Pristinamycin	-
Terbinafine	-
Ticarcillin/Clavulanate	S
TMP-SMX (Bactrim)	R
TMP-SMX (Bactrim) PO	R
TMP-SMX (Bactrim)	-
Tobramycin	-
Tolnaftate	-
Vancomycin	-
Voriconazole	-

Antibiotic Table Legend:
-: Not first choice [gray dash]
S: Sensitive [blue]
S^1: Sensitive (very effective) [green]
S^2: Sensitive (no other alternative) [orange]
S^3: Sensitive (do not use for pneumonia) [pink]
R: Treatment option that may be affected by the detected resistance marker. [Red]

Urinary Tract Infectious Disease Pathogens

Organism	Results	Est. Microbial Load*	Normal Reference Range
Candida albicans, glabrata, parapsilosis, tropicalis	Not Detected	Negative	Not Detected
Chlamydia trachomatis	Not Detected	Negative	Not Detected
Citrobacter freundii/braakii	Not Detected	Negative	Not Detected
Citrobacter koseri	Not Detected	Negative	Not Detected
Enterococcus faecium, faecalis	Not Detected	Negative	Not Detected
Escherichia coli	Not Detected	Negative	Not Detected
Gardnerella vaginalis	Not Detected	Negative	Not Detected
Klebsiella pneumoniae/oxytoca	Detected	High	Not Detected
Mycoplasma genitalium	Not Detected	Negative	Not Detected
Neisseria gonorrhoeae	Not Detected	Negative	Not Detected
Proteus mirabilis, vulgaris	Not Detected	Negative	Not Detected
Pseudomonas aeruginosa	Not Detected	Negative	Not Detected
Serratia marcescens	Not Detected	Negative	Not Detected
Staphylococcus (coagulase negative: epidermidis, haemolyticus, lugdunensis, saprophyticus)	Not Detected	Negative	Not Detected
Staphylococcus aureus	Not Detected	Negative	Not Detected

UTI PCR Report

UTI 5, UTI 5 8/7/1986 Accession ID: 250590120

Organism	Results	Est. Microbial Load*	Normal Reference Range
Staphylococcus saprophyticus	Not Detected	Negative	Not Detected
Streptococcus agalactia (group B)	Not Detected	Negative	Not Detected
Streptococcus pyogenes	Not Detected	Negative	Not Detected
Trichomonas vaginalis	Not Detected	Negative	Not Detected
Ureaplasma urealyticum	Not Detected	Negative	Not Detected
Acinetobacter baumannii	Not Detected	Negative	Not Detected

Antibiotic Resistance

Resistance Gene(s)	Antibiotic Class	Results	Est. Microbial Load*	Normal Reference Range
Class A ß-lactamase; blaKPC	N/A	Detected	N/A	Not Detected
Class A ß-lactamase; CTX-M-Group1	N/A	Not Detected	N/A	Not Detected
Class B metallo-ß-lactamase; blaNDM	N/A	Not Detected	N/A	Not Detected
Class D oxacillinase OXA-48	N/A	Not Detected	N/A	Not Detected
Class D oxacillinase OXA--51	N/A	Not Detected	N/A	Not Detected
dfr (A1, A5), sul (1,2) probes (Sulfamethoxazole and trimethoprim)	N/A	Detected	N/A	Not Detected
ermB, C; mefA	N/A	Not Detected	N/A	Not Detected
IMP, NDM, VIM Groups (Carbapenem)	N/A	Not Detected	N/A	Not Detected
MRSA* Mec-A gene	N/A	Not Detected	N/A	Not Detected
PER-1/VEB-1/GES-1 Groups (ESBL)	N/A	Not Detected	N/A	Not Detected
qnrA1, A2, B2	N/A	Not Detected	N/A	Not Detected
qnrB	N/A	Not Detected	N/A	Not Detected
qnrS	N/A	Not Detected	N/A	Not Detected
tetB,tetM	N/A	Not Detected	N/A	Not Detected
VanA, VanB (Vancomycin)	N/A	Not Detected	N/A	Not Detected
ACT, MIR, FOX, ACC Groups (Beta Lactams)	N/A	Not Detected	N/A	Not Detected

Analyzed By: Andrea Fragapane

Date: 2/28/2025

Disclaimer: This test was developed and its performance characteristics determined by Alaska Urology @ Providence Laboratories.It has not been cleared or approved by the US Food and Drug Administration(FDA).FDA does not require this test to go through premarket FDA review.This test is used for clinical purposes. It should not be regarded as investigational or for research.This laboratory is certified under the Clinical Laboratory Improvement Amendments(CLIA) as qualified to perform high complexity clinical laboratory testing.
