# Supplementary material for: Genotype→Phenotype Concordance and Ct-Informed Predictive Rules for Antimicrobial Resistance in Adult Patients with Complicated Urinary Tract Infections: Clinical and Stewardship Implications from the NCT06996301 Trial
Source: Diagnostics (Basel). 2025 Nov 21;15(23):2945. doi: 10.3390/diagnostics15232945 (PMC12691348; doi:10.3390/diagnostics15232945)
Supplement: Supplementary file 1 [file diagnostics-15-02945-s001.zip › Urine Culture.pdf]

Status Active PolicyStat ID 15968603

Origination Date	09/2014	Owner	Diana Wilkerson: Dept Director
Last Approved	06/2024	Policy Area	Laboratory Microbiology
Effective	06/2024		
Last Revised	06/2024		
Next Review	06/2026		

Urine Culture

COPY

PRINCIPLE:

Cultures from the Urinary tract can be submitted for identification of aerobic bacteria.

NOTE: Suprapubic collections may be received in the lab with the needle attached so extreme caution must be exercised.

SPECIMENS:

CLEAN CATCH: The patient is given a sterile clean catch midstream collection kit that contains towelettes. The patient is given instructions on collection; The patient is to use the first towelette to cleanse the genital area thoroughly. The patient is instructed to begin urination into the commode. After a brief period of urination the patient is instructed to stop urination and use the second towelette to again thoroughly cleanse the genital area. The patient should begin urination into the sterile container. When partially full the container may be set aside and the patient should finish urinating into the commode. The last towelette is to clean the genital area and/or hands prior to placing the lid on the collection container.

CATHERIZATION:

This is an invasive procedure and is performed by qualified nursing personnel (not laboratory staff). Indwelling catheters are clamped off for a short period of time by nursing personnel. The aspiration port is thoroughly cleaned prior to sampling. A minimum of a few mL is necessary for a culture. The clamp is released.

SUPRAPUBIC ASPIRATION:

This invasive procedure will be performed by a Physician. The specimen is labeled, orders are written and the sample and orders are transported to the lab as soon as possible.

SUPPLIES/REAGENTS:

Bi plate (if a bi plate is unavailable,use Blood agar and MacConkey agar.) 1 uL Sterile loops (if 10 uL loop is used, multiply count by an factor of 100. Be sure to indicated on the label that the 10 uL was used to streak the BAP plate).

1. The specimen is received in the lab and labels are generated using the computer.

2. Label the bi plate with the correct patient label.

3. For the BAP side, a sterile 1ul loop is used to streak down the middle and then the loop is used to streak the plate in colony count manner (at a 90 degree angle to original streak).

4. The MacConkey side is streaked for isolation.

5. Place all plates in the incubator.

6. Plates are examined daily for pathogens for up to 2 days.

Refer to the ASM Handbook for guidance.

RESULTS:

NEGATIVE RESULTS WILL BE REPORTED USING THE FOLLOWING FORMAT:

1. No Growth at 24 hours (preliminary)

2. No Growth at 48 hours (final)

Cultures showing growth will follow these guidelines:

Catheter collected, suprapubic , kidney and nephrostomy specimens , as well as pediatric patient specimens have a more stringent algorithm when working up cultures. Refer to ASM Handbook for guidance. (Generally, most uropathogenic organisms will be worked up.)

For Voided urines, the general rule is if there are 3 or more organisms , the urine is reported as contaminated. This is up to the technicians judgement based on factors such as amount of each organism present, the results of the urinalysis , the patient's information / diagnosis, etc.

For contaminated urines , the Cerner(LIS) codes used for reporting are 3DC1 (3 or more organisms present, possible contamination.) and 3DC2 (Suggest recollection if patient symptomatic.)

For pathogenic organisms worked up , use the following format:

(Colony count) (organism)

If applicable, MIC breakpoint report is included.

COPY

There are many variables which can affect the reporting of urine cultures. Techs must use their good judgement. Refer to ASM Handbook for guidance.

REFERENCES:

1. Koneman, E. W., Color Atlas and Textbook of Diagnostic Microbiology, J. B. Lippincott Co., Philadelphia, PA. p.37-38.

Approval Signatures

Step Description	Approver	Date
Director Review	Harry Latham MD: Pathologist	06/2024
Manager Review	Zack Alexander: Lab Operations Lead	06/2024
Dept Director	Diana Wilkerson: Dept Director	06/2024

COPY
