# Supplementary material for: Genotype→Phenotype Concordance and Ct-Informed Predictive Rules for Antimicrobial Resistance in Adult Patients with Complicated Urinary Tract Infections: Clinical and Stewardship Implications from the NCT06996301 Trial
Source: Diagnostics (Basel). 2025 Nov 21;15(23):2945. doi: 10.3390/diagnostics15232945 (PMC12691348; doi:10.3390/diagnostics15232945)
Supplement: Supplementary file 1 [file diagnostics-15-02945-s001.zip › UTI Panel_merged.pdf]

Bacteria
Citrobacter freundii/braakii
Gardnerella vaginalis
Staphylococcus (coagulase negative: epidermidis, haemolyticus, lugdunensis, saprophyticus)
Acinetobacter baumannii
Klebsiella pneumoniae/oxytoca
Staphylococcus aureus
Streptoccoccus agalactia (group B)
Escherichia coli
Serratia marcescens
Staphylococcus saprophyticus
Proteus mirabilis, vulgaris
Citrobacter koseri
Pseudomonas aeruginosa
Streptococcus pyogenes
Enterococcus faecium, faecalis

Fungi
Candida albicans, glabrata, parapsilosis, tropicalis

STI
Mycoplasma genitalium
Ureaplasma urealyticum
Neisseria gonorrhoeae
Trichomonas vaginalis
Chlamydia trachomatis

Resistance Genes
PER-1/VEB-1/GES-1 Groups (ESBL)
VanA, VanB (Vancomycin)
IMP, NDM, VIM Groups (Carbapenem)
Class A ß-lactamase; CTX-M-Group1
Class D oxacillinase OXA--51
qnrA1, A2
tetB, tetM
ACT, MIR, FOX, ACC Groups (Beta Lactams)
Class B metallo-ß-lactamase; blaNDM
dfr (A1, A5), sul (1,2) probes (Sulfamethoxazole and trimethoprim)
qnrS
MRSA* Mec-A
Class A ß-lactamase; blaKPC
Class D oxacillinase OXA-48
ermB, C; mefA
qnrB

1. **Purpose:** This procedure defines the responsibilities and describes the process used for extracting and purifying Total Nucleic acid from clean catch urine samples using the MagMAX™ Viral/Pathogen Ultra Total Nucleic Acid Kit on KingFisher Duo Prime and then running on the Thermo Fisher QuantStudio 7 Flex. TaqMan® Array Card is an efficient, easy-to-use system for the characterization of key microbial targets. This panel includes TaqMan® assays that have been optimized for the detection of 28 organisms, which include bacterial, fungal and parasitic targets along with antibiotic resistance to 16 drugs. Control assays i.e. TaqMan® Universal Extraction Control Organism, and TaqMan® Amplification controls are included to track the overall quality of the entire process.
2. **Materials:**
   - 2.1. Components of the MagMax™ Viral/Pathogen Ultra Nucleic Acid Isolation Kit (Cat# A42356)- stored at 15℃ to 25℃
     - 2.1.1. Binding Solution 53 mL
     - 2.1.2. Wash Buffer 100 mL
     - 2.1.3. Elution Solution 10 mL
     - 2.1.4. Proteinase K 1 mLTotal Nucleic Acid Binding Beads 2 mL
     - 2.1.5. Enzyme mix 5 mL (stored at -15℃ to -25℃)
   - 2.2. TaqPath 1-Step RT-qPCR Master Mix -15℃ to -25℃
   - 2.3. KingFisher™ deep-well 96 plate
   - 2.4. Duo Prime 12-Tip comb for use in deep well 96 plate
   - 2.5. KingFisher™ Duo Elution strip and cap
   - 2.6. UTI TaqMan card (2℃ to 8℃)
3. **Specimens:**
   - 3.1. Specimens should be collected and transported as per the established SOP.
   - 3.2. Specimens can be stored refrigerated (2-6°C) for up to 48 hours. If testing is prolonged, the sample can be stored frozen for up to one month.
   - 3.3. Any remaining stock nucleic acid will be stored at -15°C to -25°C for a minimum of one month.
4. **Quality Control:**
   - 4.1. TaqMan® Universal Extraction Control Organism serves as a process control for the DNA extraction and purification process and is processed throughout the entire PCR workflow. TaqMan® Universal Extraction Control Organism is Bacillus atrophaeus, a gram-positive bacteria whose structural characteristics make it an ideal control for sample extraction and purification. (stored at -15℃ to -25℃)
   - 4.2. TaqMan® Urinary Tract Microbiota Amplification Control contains a linearized multi-target plasmid with target sequences for each available urinary tract microbiota profiling assay. It can be included in profiling experiments as a positive control and for troubleshooting. It will be included on the first UTI card run weekly as a stand-alone

sample at real-time PCR and carried through the remainder of the workflow. (stored at -15℃ to -25℃)

4.3. Negative Control is well that remains empty throughout the DNA extraction procedure, to which Nuclease-Free Water is added directly to the elution plate following the extraction and will be used in the Taq card to check for any possible carryover.

5. Setting up the 96 Deep Well Sample Plate

5.1. Take the Enzyme mix and *Bacillus atrophaeus* out of the freezer.
5.2. Invert the Patient's Urine Sample 10X or swirl for 10 sec.
5.3. Label microfuge tube for corresponding urines.
5.4. Aliquot 1000 uL of urine to the appropriate microfuge tube and spin for 5 mins at 9000 RPM.

While waiting for urines to spin down, Steps 5.5-5.7 can be completed.

5.5. Add 1000 uL of Wash Buffer in Row C of 96 Deep Well Sample Plate for every sample that will be tested.
5.6. Add 1000 uL of 80% Ethanol to Row E of 96 Deep Well Sample Plate for every sample that will be tested.
5.7. Add 500 uL of 80% Ethanol to Row G of 96 Deep Well Sample Plate for every sample that will be tested.
5.8. Decant Urine by pipetting ~900 uL from the microfuge tube down to the 0.1 uL mark so as to not disturb the pellet and discard along with the pipette tip.
5.9. Add 400 uL PBS (Phosphate Buffered Saline) to each microfuge tube.
5.10. Vortex each microfuge tube with urine/PBS sample for 10-20 seconds to resuspend the pellet.
5.11. Add the following to Row A of 96 Deep Well Sample Plate for every sample that will be tested:
    5.11.1. 50 uL Enzyme Mix - Pipette slowly, highly viscous.
    5.11.2. 10 uL B. atrophaeus control - change tip between each well.
    5.11.3. 400 uL of urine suspended in PBS - change tips between each well/sample. Discard tubes after being transferred to wells.
5.12. Put a new Tip Comb in 96 Deep Well Sample Plate Row H.

Plate Row	Reagent	Volume per Well
**A**	**Sample**	**~ 500 uL**
B	Empty	
**C**	**Wash Buffer**	**1,000 uL**
D	Empty	
**E**	**80% Ethanol**	**1,000 uL**
F	Empty	
**G**	**80% Ethanol**	**500 uL**
H	12 Tip Comb	

5.13. Load 96 Deep Well Sample Plate onto KingFisher with the A1 cell in the corresponding position on KingFisher.
5.14. Add 100 uL of Pathogen Elution Solution to the Elution Strip for every sample that will be tested.

Plate Row	Reagent	Volume per Well
**A**	**Elution Solution**	**100 uL**

5.15. Select MVP_Ultra_Duo and Press Start □
5.16. Follow the prompts on the screen and then press the ✔.

While waiting for the 1st part of the KingFisher run, steps 5.17 - 5.20 can be done in preparation.

5.17. Put Enzyme Mix and *B. atrophaeus* back into the freezer.
5.18. In a 50mL conical tube, add 530 uL of Binding Solution per sample tested plus two extra.
5.19. In the same 50mL conical tube, add 20 uL of binding Beads per sample tested plus two extra. Vortex the binding beads before adding them to the solution.
5.20. Invert or swirl the tube with Binding Bead Solution to mix, DO NOT VORTEX!
5.21. After ~20 minutes the KingFisher will ask for Binding Bead Solution and Proteinase K.

5.22. Remove the 96 Deep Well Sample Plate from the KingFisher.

5.23. Add 10 uL of Proteinase K to Row A of 96 Deep Well Sample Plate for every sample that will be tested.

5.23.1. Change the pipette tip if the tip comes in contact with the inside of the sample well.

5.24. Add 550 uL of Binding Bead Solution (invert or swirl before use) to Row A of 96 Deep Well Sample Plate for every sample that will be tested.

5.24.1. Change the pipette tip if the tip comes in contact with the inside of the sample well.

5.25. Load the 96 Deep Well Sample Plate back onto the KingFisher Duo and press the ✔.

5.26. Take the UTI TaqMan card out of the refrigerator and Master Mix out of the freezer to bring it to room temperature.

6. Preparing TaqMan card for analysis on QuantStudio 7 Flex:

6.1. Label microfuge tubes to correspond with the samples being tested.

6.2. Add the following to each microfuge tube:

6.2.1. 32 uL of UltraPure DI H2O

6.2.2. 28 uL of 1-Step RT-qPCR Master Mix (DO NOT VORTEX!)

6.3. Label one microtube **NTC** and add the following:

6.3.1. **82 uL** of UltraPure **DI H2O**

6.3.2. **28 uL** of 1-Step RT-qPCR **Master Mix** (DO NOT VORTEX!)

6.4. After the extraction on the KingFisher is complete ~30 minutes, discard the 96 Deep Well Sample Plate in BioHazard.

6.5. Take the elution strip off the KingFisher and transfer 50 uL of elution into the corresponding microfuge tube.

6.5.1. Discard the pipette tip after each sample.

6.5.2. Discard the elution strip after all samples have been transferred.

6.6. Mix sample 5-6 times with pipette fill port on UTI TaqMan card with 100 uL of the corresponding sample from microfuge tube and discard tube.

6.6.1. Dispense samples slowly and steadily so as not to introduce bubbles.

6.7. Fill the 8th port on the UTI TaqMan card with 100 uL UltraPure DI H2O - to be used as an NTC (No Template Control).

6.8. Centrifuge TaqMan card at 1200 rpm for 1 minute.

6.9. Repeat step 6.7.

6.9.1. If the card appears to have any bubbles, you can repeat step 6.7 again, but DO NOT centrifuge the card more than 3 times.

6.9.2. Seal the card using the TaqMan Array Card Sealer by only pushing forward.

6.9.2.1. DO NOT pull the handle back towards you!

6.9.3. Cut off the foil tip near the edge of the plastic where the open ports were.

7. Loading Card and Running Experiment on QuantStudio 7 Flex:

7.1. Open Door on Flex (can be done in two different ways).
 7.1.1. Press Red Up Arrow on the screen of Flex.
 7.1.2. From QuantStudio software: Select Console and choose “Open Door” on the Top Task Bar.
7.2. Load the Sealed TaqMan card onto the arm with A1 in the top left corner and the barcode facing you.
7.3. Shut the door on Flex using either of the two ways mentioned above.
7.4. Select “New Experiment” from the top left drop-down menu → From Template
 7.4.1. Choose Office Template Folder
 7.4.2. Select Custom UTM Template and open.

Experiment Type: Array Card, Experiment: Comparative Ct, Run Type: Fast, Import the plate file for card, change the run method settings to the following...				
**Step**	**Stage**	**Cycles**	**Temp.**	**Time**
UNG incubation	1	1	25℃	2 minutes
Reverse Transcription	2	1	50℃	15 minutes
Polymerase Activation	3	1	95℃	2 minutes
Amplification	4	40	95℃	3 seconds
			60℃	30 seconds

7.5. On Q12 Software Experiment Menu, choose Define under the setup option.
 7.5.1. Change Sample Names to correspond with the samples being tested on the card.
7.6. Go to Run.
 7.6.1. The Green Drop Down Menu “Start Run”
 7.6.2. Choose Console.
 7.6.3. Choose the folder to save the run to and assign the name run following lab standards.
7.7. Go back to the Green Drop Down Menu “Start Run”
 7.7.1. Choose Console and your run will start.
7.8. ~1 hour 30 minutes, the experiment will be over. Unload the used card following steps 7.1 - 7.3 except removing the card and discarding it in the BioHazard waste bin.

8. References:

8.1. Urinary Tract Microbiota Profiling Experiments: APPLICATION GUIDE TaqMan® Fast Virus 1-Step Master Mix.

8.2. MagMAX™ Viral/Pathogen Ultra Total Nucleic Acid Extraction - KF Duo Prime Protocol EA.

Version	Date	Reason for Revision	Person Making Revision
1.0	10/1/2021	Original Release	NA

1. **Purpose:** The purpose of this procedure is to standardize how to interpret positive and negative results for all assays run using the QuantStudio 7 Flex software.
2. **Materials:**
   2.1. QuantStudio 7 Software
   2.2. TrueMed LIS
3. **Procedure:**
   3.1. The following Ct ranges are used for reporting all pathogens.
      3.1.1. **Critically High:** 10.000 - 21.000
      3.1.2. **High:** 22.000 - 26.000
      3.1.3. **Medium:** 27.000 - 28.000
      3.1.4. **Low:** 29.000 - 32.000
      3.1.5. **Negative:** 40.000 - 33.000; 09.000 - 1.000
   3.2. The following Ct ranges are used for reporting all resistance genes.
      3.2.1. **Positive:** 10.000 - 32.000
      3.2.2. **Negative:** 33.000 - 40.000; 1.000 - 9.000
   3.3. In order to produce a valid result for the report, you will need to take into account the C_t value generated, the amplification plot graph, and the multicomponent plot graph if the amplification graph is unclear.
      3.3.1. **Amplification:** Record pathogen/resistance gene as positive if it contains C_t value and amplification (example below shows E.Coli with a C_t value of 25.2 and amplification)

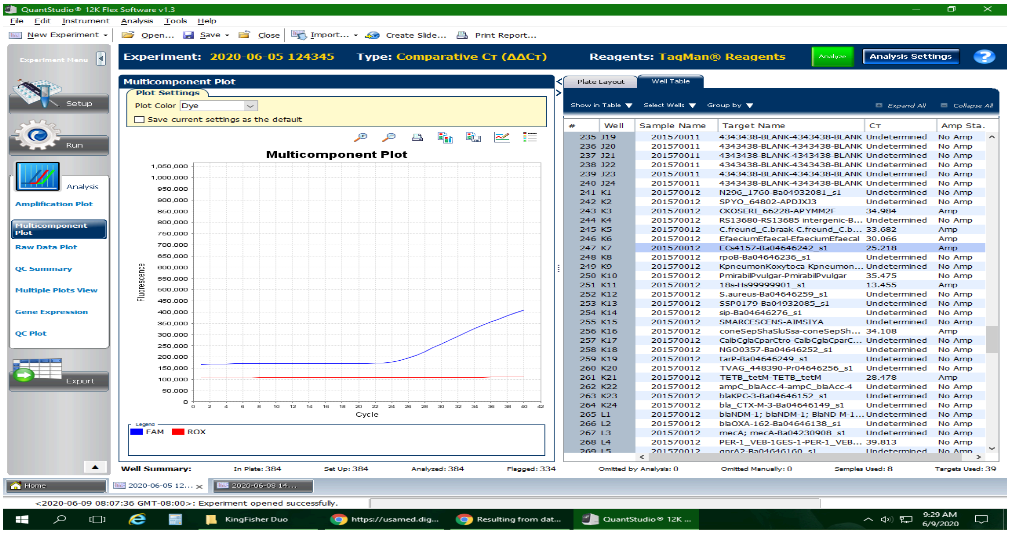

3.3.2. Record the pathogen/resistance gene as negative if the Ct value is undetermined (whether amplification is present or absent).

3.3.3. Record pathogen/resistance gene as negative if Ct value is present and there is no amplification (example below shows Proteus mirabilis, vulgaris Ct value of 35.4 and no amplification).

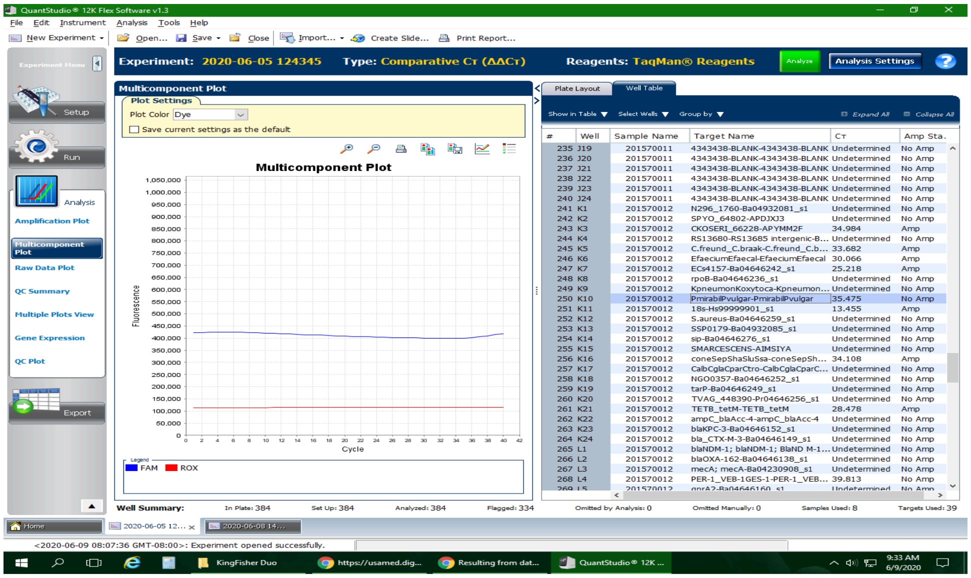

3.3.4. Check the amplification and multicomponent plot graphs for each target containing a Ct value and the amplification status is inconclusive.

3.3.4.1. If the graph shows evidence of amplification, record the pathogen/resistance gene as positive/negative based on the Ct value. If you cannot conclude that there is amplification, record the pathogen/resistance gene as negative.

3.3.5. To rule out any false positives, if the Ct value for a given pathogen/resistance gene is over 30 and the curve on the amplification plot starts after cycle 32, report the pathogen/resistance gene as negative.

3.3.5.1. If the Ct value for a pathogen/resistance gene is over 30 and the curve on the multicomponent plot is amplified after cycle 30, report the pathogen/resistance gene negative.

3.3.6. **Amp Score:** If there is an Amp Score of 1.2 or greater, amplification and a curve seen on the amplification plot or multicomponent plot, then record the pathogen/resistance gene as positive. If the Amp Score is less than 1.2, amplification and a curve seen on the amplification plot or multicomponent plot

record the pathogen/resistance gene as positive. If the Amp Score is less than 1.2, the amplification and **NO** curve seen on the amplification plot or multicomponent plot record the pathogen/resistance gene as negative.

3.3.7. **CQ Conf:** The CQ conf score is an additional confirmatory quantitative measure that can determine the presence or absence of a pathogen/resistance gene. If criteria for C_t, and Amp Score are met, the CQ conf can be used for confirmation. If the CQ conf is 0.90 or greater, record the pathogen/resistance gene as positive.

4. Quality Controls:

4.1. The *B. atrophaeus* control must have a Ct value within our reportable range and amplification before you can report out that sample.

Version	Date	Reason for Revision	Person Making Revision
1.0	10/01/2021	Original Release	NA
